# Reconciling fossils with phylogenies reveals the origin and macroevolutionary processes explaining the global cycad biodiversity

**DOI:** 10.1111/nph.19010

**Published:** 2023-06-11

**Authors:** Mario Coiro, Rémi Allio, Nathan Mazet, Leyla J. Seyfullah, Fabien L. Condamine

**Affiliations:** ^1^ Department of Palaeontology University of Vienna 1090 Vienna Austria; ^2^ Ronin Institute for Independent Scholarship Montclair NJ 07043 USA; ^3^ Centre de Biologie pour la Gestion des Populations, INRAE, CIRAD, IRD, Montpellier SupAgro Université de Montpellier 34988 Montpellier France; ^4^ CNRS, Institut des Sciences de l'Evolution de Montpellier, Université de Montpellier Place Eugène Bataillon 34095 Montpellier France

**Keywords:** Antarctica, Cycadales, fossil leaves, Greenland, historical biogeography, total‐evidence analysis

## Abstract

- The determinants of biodiversity patterns can be understood using macroevolutionary analyses. The integration of fossils into phylogenies offers a deeper understanding of processes underlying biodiversity patterns in deep time. Cycadales are considered a relict of a once more diverse and globally distributed group but are restricted to low latitudes today. We still know little about their origin and geographic range evolution.
- Combining molecular data for extant species and leaf morphological data for extant and fossil species, we study the origin of cycad global biodiversity patterns through Bayesian total‐evidence dating analyses. We assess the ancestral geographic origin and trace the historical biogeography of cycads with a time‐stratified process‐based model.
- Cycads originated in the Carboniferous on the Laurasian landmass and expanded in Gondwana in the Jurassic. Through now‐vanished continental connections, Antarctica and Greenland were crucial biogeographic crossroads for cycad biogeography. Vicariance is an essential speciation mode in the deep and recent past. Their latitudinal span increased in the Jurassic and restrained toward subtropical latitudes in the Neogene in line with biogeographic inferences of high‐latitude extirpations.
- We show the benefits of integrating fossils into phylogenies to estimate ancestral areas of origin and to study evolutionary processes explaining the global distribution of present‐day relict groups.

## Abstract 2

This article is part of the Special Collection ‘Global plant diversity and distribution’. See https://www.newphytologist.org/global-plant-diversity for more details.

## Introduction

Extinction is an important process shaping biodiversity. Given the pervasiveness of extinction in deep time, comparative studies of living organisms offer a limited window on evolutionary patterns and processes, especially when looking at geological timescales (Crisp *et al*., 2011; Marshall, 2017). In the past three decades, an extended toolbox has been developed for macroevolutionary analyses of dated phylogenies (Nee *et al*., 1992, 1994; Morlon, 2014). However, even if some neontological approaches seem to be robust in the absence of extinct lineages (Morlon *et al*., 2011; Beaulieu & O'Meara, 2015; Condamine *et al*., 2020), studies have shown that integrating fossil information can greatly improve macroevolutionary inferences (Slater *et al*., 2012; Fritz *et al*., 2013; Hunt & Slater, 2016; Oliveros *et al*., 2020). Recognition of the fundamental importance of fossils in macroevolutionary studies has led to the development of new methods that use the fossil record to infer diversification dynamics (Silvestro *et al*., 2014, 2018a; Mitchell *et al*., 2019), as well as methods to intertwine fossils in molecular phylogenies (Zhang *et al*., 2016; Gavryushkina *et al*., 2017). This has led to a modest fossil renaissance in macroevolution (King *et al*., 2017; Slater *et al*., 2017), which may have important implications in our understanding of the geographic patterns and processes underlying global diversity and distribution, especially for ancient groups like plants (Crisp *et al*., 2011; Mao *et al*., 2012; May *et al*., 2021).

Despite the expansion of statistical methods, the plant fossil record remains particularly problematic and yet central to macroevolutionary analyses. Different plant organs tend to fossilize separately (Bateman & Hilton, 2009), and the phylogenetic informativeness of plant fossils varies between organ types (Bateman & Simpson, 1998; Coiro & Barone Lumaga, 2018; Coiro *et al*., 2020a) and conservation modes, as well as within and between different clades. This complicates both the taxonomic assignment of plant fossils and the estimate of stratigraphic ranges. However, this has not discouraged people from using generic‐level occurrences to infer macroevolutionary dynamics at different scales (vascular plants: Silvestro *et al*., 2015; ferns: Lehtonen *et al*., 2017; conifers: Condamine *et al*., 2020). Even if such investigations are probably robust, the possibility of systematic error introduced by misinterpretation of the fossil record cannot be excluded. Integrating plant fossils during phylogenetic reconstruction represents an alternative. Total‐evidence dating (Ronquist *et al*., 2012) with the fossilized birth‐death (FBD) model (Heath *et al*., 2014; Zhang *et al*., 2016) has been used to estimate both phylogenetic relationships and divergence times in many fossil‐rich plant groups (Grimm *et al*., 2015; Larson‐Johnson, 2016; Renner *et al*., 2016; May *et al*., 2021; Zhang *et al*., 2022). These analyses take advantage of well‐preserved fossils or well‐understood relationships between fossil and extant taxa to improve phylogenetic and divergence time inferences.

Among plants, cycads (order Cycadales) include > 360 extant species (Govaerts *et al*., 2021) of which 68% are threatened with extinction (IUCN, 2022). Originating in the Paleozoic (Hermsen *et al*., 2006; Condamine *et al*., 2015), cycads are widely considered quintessential ‘living fossils’, due to their supposed long‐term morphological stasis and their apparent dominance in the fossil record followed by a decline toward the present. Indeed, cycad evolution was thought to have reached its pinnacle in the Middle Jurassic, with a subsequent decline in morphological and taxonomic diversity (Niklas *et al*., 1983), possibly driven by competition with the angiosperms (Norstog & Nichols, 1997) or the decline of nonavian dinosaurs (Mustoe, 2007; Butler *et al*., 2009). This decline is thought to have led to a depauperate modern cycad flora (Harris, 1961). The idea of cycad diversity as a relic from the Mesozoic has been challenged on molecular grounds, since the extant species diversity is inferred to be the result of Miocene‐Pleistocene radiations in most genera (Treutlein & Wink, 2002; Nagalingum *et al*., 2011; Salas‐Leiva *et al*., 2013; Condamine *et al*., 2015; Liu *et al*., 2022). Despite this, the old ‘living fossil’ viewpoint still continues to influence some views on the evolution of the group (Zhang *et al*., 2015; Nackey *et al*., 2018), its biotic interactions in deep time (Cai *et al*., 2018; Salzman *et al*., 2020), and its geographic origin and range expansion (Salas‐Leiva *et al*., 2013).

Our poor understanding of the cycad fossil record hinders testing hypotheses about their macroevolutionary trajectories and especially their historical biogeography. The relationships of the main Mesozoic taxa are still poorly understood (Hermsen *et al*., 2006; Coiro & Pott, 2017), and it remains unclear whether many genera represent biological rather than purely taxonomic units (Pott *et al*., 2007). Fossil cycads representing reproductive structures are rare and have been often overlooked or have little or unclear phylogenetic value, though better‐preserved specimens have led to fruitful insights (Spencer *et al*., 2017; Rothwell *et al*., 2022; Elgorriaga & Aktinson, 2023). Although the leaf record is much more abundant, it is also poorly understood. Recently, the cycadalean nature of one of the most common leaf taxa from the Mesozoic, *Nilssonia* Brongniart, has been questioned on chemical grounds (Vajda *et al*., 2017). Similarly, the Early Cretaceous leaf *Mesodescolea* S.Archang., thought to be the closest relative of extant *Stangeria* Hook. ex Hook.f., has been reinterpreted as an angiosperm leaf (Coiro *et al*., 2020b). Even if some of the extant genera start to be recognized in sediments as old as the Eocene (Hill, 1978; Carpenter, 1991; Kvaček, 2002; Su *et al*., 2014; Erdei *et al*., 2018), many Cenozoic leaf fossils defy any attempt of classification into the extant cycad groups, such as *Eostangeria* Barthel from the Paleocene of North America and Europe, Eocene of Germany, and Miocene of Bulgaria (Barthel, 1976; Kvaček & Manchester, 1999; Uzunova *et al*., 2001), and *Pseudodioon* Erdei, Agkun et Barone Lumaga from the Miocene of Turkey (Erdei *et al*., 2009). Moreover, the Mesozoic genus *Ctenis* Lindl. & Hutt. is present in the Eocene of Oregon (Erdei & Manchester, 2015), a locality where cycads are absent today. Likewise, *Dioonopsis* Horiuchi & Kimura, with three species from the Paleocene of Japan and Eocene of North America (Horiuchi & Kimura, 1987; Erdei *et al*., 2012), has been linked with the extant genus *Dioon* Lindl. based on general leaf morphology (Moretti *et al*., 1993) as well as phylogenetic analyses of anatomy and morphology (Hermsen *et al*., 2006; Martínez *et al*., 2012), but this link has been recently questioned (Barone Lumaga *et al*., 2015; Erdei & Manchester, 2015; Erdei *et al*., 2019). Given the age of cycads and long branches subtending radiations of extant genera, the uncertainties in fossil placements hinder any macroevolutionary inferences that would be based solely on dated phylogenies (Crisp *et al*., 2011).

Few phylogenetic analyses have included fossil cycads (Hermsen *et al*., 2006; Martínez *et al*., 2012). Although these pioneering works have merits, they still present many issues. First, the inferred topology is invariably incompatible with the topology of extant cycads obtained using molecular data, a conflict that calls into question the relationship retrieved between fossil and extant taxa (Coiro & Pott, 2017). Second, these analyses were conducted using generic‐level taxa, even though paleobotanical practice may lead to the creation of heterogeneous, nonmonophyletic genera for practical use (Harris, 1961). Third, they included genera with uncertain cycadalean affinities, such as the seed fern‐like *Ticoa* S.Archang. and *Kurtziana* Frenguelli, or the angiosperm *Mesodescolea* S.Archang. (Coiro *et al*., 2020b). Fourth, they included both leaf and stem taxa, each scored for only a few nonoverlapping and limited characters, which can create conflicting phylogenetic signals that cannot be disentangled.

**Fig. 1:**
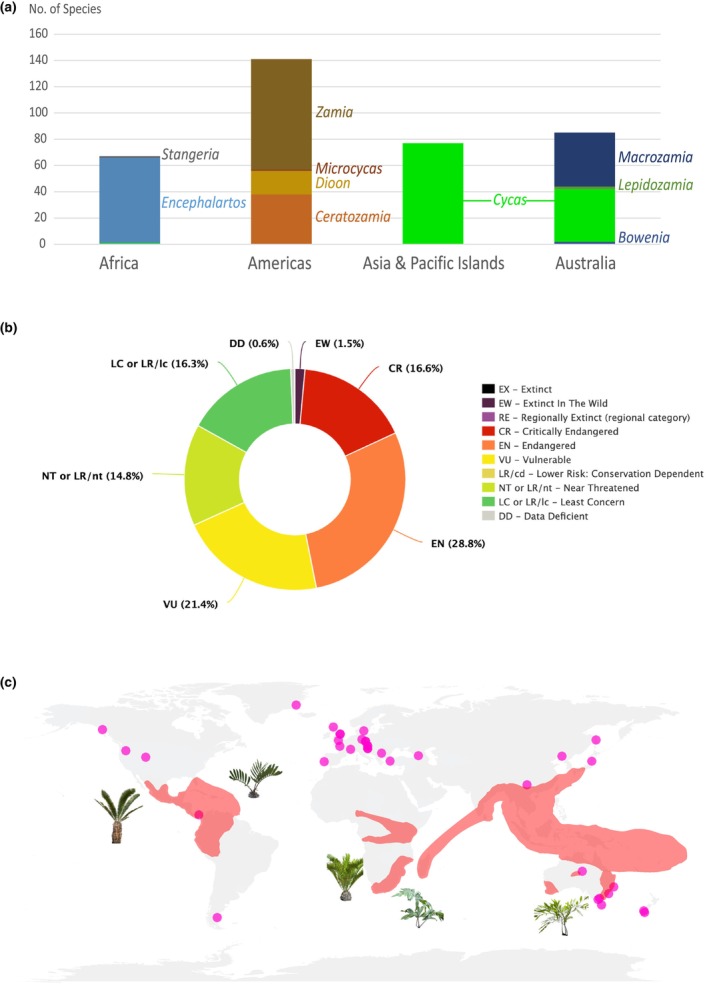
Global distribution of Cycadales. (a) Species richness of the 10 extant cycad genera across continents. (b) IUCN threat categories for 337 extant cycad species (redrawn from IUCN, 2022). (c) Map showing the extant distribution of cycad diversity (red areas) and sampled fossil localities (purple dots), indicating broader geographic distribution in the past.

Here, we study the macroevolutionary processes underlying the assembly of global diversity and distribution of cycads by reconciling fossils and phylogeny through Bayesian total‐evidence analysis. Based on morphological analyses of leaves, 60 fossil cycad species were integrated in a single phylogenetic inference performed with *c*. 87% of all extant species for which both morphological and molecular data were collected. The extant cycad distribution is highly disjunct today with sister genera located on different continents (Fig. 1), such as *Encephalartos* Lehm. (Africa) and *Lepidozamia* Regel (Australia) or *Zamia* L. + *Microcycas* A.DC. (Americas), and *Stangeria* (Africa). We assess the roles of dispersal, extinction, and vicariance in establishing the current cycad distribution by inferring the ancestral geographic origin and tracing the historical biogeography with a time‐stratified and tectonic‐informed process‐based model. We also tested the effect of treating fossil ranges as fundamentally ambiguous by scoring absences as unknown. Given their appearance in the fossil record in the Paleozoic and limited dispersal ability, a Pangean origin and vicariance events during the breakup of ancient supercontinents are expected. Moreover, the latitudinal span of extant cycads is currently restricted to subtropical latitudes, but fossils indicate wider latitudinal distribution in the Mesozoic (Harris, 1926; Smoot *et al*., 1985; Hermsen *et al*., 2006; Fig. 1), suggesting high‐latitude extirpations (Meseguer & Condamine, 2020). We finally infer the latitudinal span of cycads through time to test the hypothesis of poleward extinctions in the Cenozoic.

## Materials and Methods

### Global cycad diversity and distribution

The World Checklist of Vascular Plants (WCVP; Govaerts *et al*., 2021) lists 362 recognized species of Cycadales. However, new cycad species are regularly described each year (Calonje *et al*., 2021; Pérez‐Farrera *et al*., 2021a,b, 2022; Martínez‐Domínguez *et al*., 2022), so that there are now 370 cycad species (The World List of Cycads: http://cycadlist.org/, Calonje *et al*., 2022). From the WCVP data, we extracted the distribution range of each species (‘geographic_area’) to categorize their continental distribution for biogeographic analyses (to be described later).

### Molecular phylogenetics

We sampled 321 extant species out of 370 recognized species, including all genera and a species sampling across all genera as follows: *Bowenia* Hook. ex Hook.f. 2/2, *Ceratozamia* Brongn. 33/38, *Cycas* L. 104/118, *Dioon* 13/18, *Encephalartos* 65/65, *Lepidozamia* 2/2, *Macrozamia* Miq. 27/41, *Microcycas* 1/1, *Stangeria* 1/1, and *Zamia* 74/84. This results in 86.8% (321/370) of the total species richness. *Ginkgo biloba* L. was added as an outgroup, which is recognized as the sister lineage of cycads (Liu *et al*., 2022; Yang *et al*., 2022). The molecular matrix was assembled from sequences available on GenBank. We included 18 loci including plastidial (matK, rbcL), mitochondrial (26S), and nuclear genome sequences (AC3, F3H, GroES, RPB1, SAMS, CyAG, HTS, WRKY4, 40S, 5.8S, LiSH, GTP, PHYP, HZP, and PEX4).

We aligned the noncoding genes using Mafft 7.110 (Katoh & Standley, 2013) with the E‐INS‐i algorithm, while we aligned the coding genes using the Omm Macse pipeline (omm_macse_v11.05.sif; Ranwez *et al*., 2018), which includes nucleotides and amino acid alignment steps combined with several cleaning steps (including HMMCleaner). All the resulting alignments were checked for codon stops and eventually refined by eye with Mesquite 3.7 (Maddison & Maddison, 2021). All gene alignments were concatenated into a nucleotide supermatrix. The final molecular matrix contained 11 895 nucleotides.

We performed maximum likelihood (ML) to reconstruct phylogenetic relationships of extant species. ML inference was implemented with Iq‐Tree 2.2.0 (Minh *et al*., 2020) using ModelFinder to select the best‐fit partition scheme and the best‐fitting substitution model for each partition (*‐m MFP+MERGE*, Chernomor *et al*., 2016; Kalyaanamoorthy *et al*., 2017). For Iq‐Tree analyses, we estimated the most likely tree with 100 separate ML searches, as well as 100 searches, which after initial model optimization on a parsimony tree used 100 random tree topologies as starting trees for each search. As recommended, we optimized ML searches to avoid local optima by (1) increasing the number of unsuccessful iterations before stopping tree optimization to 500 (*‐nstop 500*), and (2) decreasing the perturbation strength for randomized NNI to 0.2 (*‐pers 0.2*). Branch supports were evaluated with 1000 ultrafast bootstraps (UFBS; Hoang *et al*., 2018), with strong UFBS values ≥ 95% considered as strong support.

### Sampling fossils and morphological data

We generated a matrix of 31 morphological characters covering leaf morphology and cuticular anatomy, based on literature and direct observations (Supporting Information Methods S1; Table S1; Notes S1). We then scored characters for 60 species of fossil cycad leaves, including macrofossils and dispersed cuticles, as well as for the extant taxa of the molecular matrix. Coding was based on observations of the specimens or descriptions and photographs of the specimens available in the literature (Greguss, 1968; Mickle *et al*., 2011; Barone Lumaga *et al*., 2015; Coiro & Pott, 2017; Vovides *et al*., 2018; Erdei *et al*., 2019; Coiro *et al*., 2020a, 2021; Glos *et al*., 2022).

### Bayesian total‐evidence analyses

Estimates of divergence times were carried out under a Bayesian total‐evidence dating approach with the FBD model, which explicitly models the speciation, extinction, fossilization, and sampling processes (Heath *et al*., 2014; Zhang *et al*., 2016; Gavryushkina *et al*., 2017). For this analysis, the outgroup (*Ginkgo biloba*) was removed, to avoid issues with the long unsampled fossil history of this lineage. Bayesian inferences were performed with MrBayes 3.2.7a, with the six molecular partitions as estimated by ModelFinder in Iq‐Tree and set to have their own evolutionary model. Models of sequence evolution were set with the reversible‐jump Markov Chain Monte Carlo (MCMC) with the gamma rate for site heterogeneity and the proportion of invariable sites (Huelsenbeck *et al*., 2004). We also set one partition for all the morphological characters. Morphological evolution was computed with the Markov‐k model (Lewis, 2001) with the correction for variable characters and a gamma‐distributed rate variation across characters. Variable gamma rates were chosen as the preferred model to analyze the different partitions. Substitution model parameters (rates, gamma shape, and invariable sites) were unlinked between partitions. Two runs of eight incrementally heated MCMC starting from a random tree were performed.

The dating was conducted using the independent gamma rates (IGR) model in which tree branches have their own evolutionary rates (equivalent to the uncorrelated lognormal relaxed clock; Drummond *et al*., 2006). The IGR clock with an exponential prior for the variance parameter was used for the molecular partitions (*prset clockvarpr = igr*) with an exponential prior on the variance of the gamma distribution from which the branch lengths are drawn in the IGR model (*prset igrvarpr = exp(10)*), while a strict clock was used for morphology. The mean clock rate (mean substitution rate per site per million years (Myr)) is assigned a lognormal prior (*prset clockratepr = lognorm(‐6*,*0.5)*); giving a mean *c*. 0.001 substitution rate per site per Myr.

Since preliminary analyses failed to converge without topological constraints, we enforced the best IQ‐TREE topology for extant species with *partial* constraints, meaning that the placement of fossil taxa is inferred while the topology of extant species remained fixed. The 60 fossil taxa were added as tips based on single specimens or multiple specimens from the same locality. We assign priors for the fossil ages based on the geological time scale. This typical step in total‐evidence dating aims at calibrating the fossil taxa instead of the internal nodes of the tree. Uncertainty about the dating of the fossiliferous localities was implemented as a uniform distribution for the age of the fossil tips, for example, *Bowenia johnsonii* R.S.Hill, K.E.Hill, Carpenter et Jordan is an early Eocene fossil that translates into *uniform(47.8*,*56)*. Sources for the fossil ages are listed in Table S2. The speciation, extinction, fossilization, and sampling processes are explicitly modelled using the FBD process: *prset brlenspr = clock:fossilization* (Heath *et al*., 2014; Zhang *et al*., 2016). The three FBD parameters were set as follows: (1) the fossilization prior with a beta distribution: *prset fossilizationpr = beta(1*,*1)*; (2) the speciation rate with an exponential prior: *prset speciationpr = exp(10)*; and (3) the relative extinction rate with a beta prior: *prset extinctionpr = beta(1*,*1)*. The sampling strategy of extant taxa was set to diversity (*prset samplestrat = diversity*) with sampling fraction set to 0.87 (*prset sampleprob = 0.87*), wherein fossils are sampled randomly and can be tips or ancestors (Zhang *et al*., 2016). A uniform prior was set on the tree age, bounded by the lowest age of the Lopingian at 259.1 million years ago (Ma) (that correspond to the older fossils assignable with confidence to the cycads) and the upper age of the Famennian at 358.9 million years ago (Ma) (that correspond to some of the oldest stem seed plants): *prset treeagepr = uniform(259.1*,*358.9)*.

The total‐evidence dating was finally run for 50 million MCMC generations with trees and associated model parameters sampled every 50 000 generations. All the analyses were carried out on the CIPRES science gateway (Miller *et al*., 2010). We performed the analyses three times to ensure repeatability of the results. Convergence diagnostics were checked for each analysis (i.e. average standard deviation of split frequencies (ASDSF) < 0.05, potential scale reduction factor (PSRF) close to 1.0) as well as the effective sample size (ESS) > 200 in Tracer 1.7.1 (Rambaut *et al*., 2018). A consensus tree was obtained after discarding 25% of the generations as burn‐in to compute posterior probability (PP), median age, and 95% highest posterior density (HPD) for each node.

### Representing uncertainty in fossil placement

We used RoguePlots (Klopfstein & Spasojevic, 2019) to investigate the uncertainty in the fossil placement of some taxa, namely the members of the two genera *Dioonopsis* Horiuchi & Kimura and *Eostangeria* Barthel. These were selected for their importance as calibrations (*Dioonopsis*) and their uncertain relationship in the literature (*Eostangeria*). From the posterior distribution of trees from the constrained analysis, we generated tree summaries to show the placement of these taxa on the consensus phylogeny.

### Estimating ancestral latitudes

The latitudinal span of the Cycadales throughout their evolutionary history was reconstructed using the directional Brownian–Motion method of Silvestro *et al*. (2018b) with the variable trend implemented in Zhang *et al*. (2022). Mean latitude for the extant genera was obtained using occurrence data obtained from the Global Biodiversity Information Facility (GBIF). These occurrences were obtained by querying the different genera using the function *occ_search* from the R package rgbif (Chamberlain *et al*., 2019), and then cleaned using the function *clean_coordinates* from the R package coordinatecleaner (Zizka *et al*., 2019). Further cleaning of occurrences was done manually. This resulted in mean latitude estimates for 207 extant taxa. Paleolatitudes for the fossil taxa were obtained using the paleolatitude calculator (van Hinsbergen *et al*., 2015). Approximate position of the fossil localities was queried, and the average age of the localities was used to obtain paleolatitudes from the model. This resulted in 48 fossils with constrained paleolatitudes. The MCMC was run for 100 000 generations using 100 trees randomly sampled from the posterior sample. Traits were rescaled by 10 to help convergence.

### Estimating historical biogeography

We estimated the ancestral areas of origin and geographic range evolution for Cycadales using the ML approach of dispersal–extinction–cladogenesis (DEC, Ree & Smith, 2008) as implemented in the C++ version (Beeravolu & Condamine, 2016), as well as the R package biogeobears (Matzke, 2018). To infer the biogeographic history of a clade, DEC requires a time‐calibrated tree (i.e. the consensus tree obtained from the Bayesian TED analysis), the current distribution of each species, a set of geographic areas, and a time‐stratified geographic model that is represented by connectivity matrices for specified time intervals spanning the entire evolutionary history of the group.

We first defined a set of geographic areas based on paleogeographic knowledge as follows: (1) West Palearctic, defined as Western Europe to the Urals, (2) East Palearctic, defined as east of the Urals, above 3000 m in the Himalayas and north of Sichuan in China, (3) West Nearctic, defined as Western North America including the Rocky Mountains, (4) East Nearctic, defined as North America east of the Rocky Mountains, (5) Central America, going from the northern border of Mexico southward to the border between Panama and Colombia, (6) Caribbean Islands, excluding Trinidad and Tobago, (7) South America, defined as all countries from Colombia to Argentina and including Trinidad and Tobago, (8) Africa, defined as the whole African continent and Arabian Peninsula but excluding the islands in the Indian Ocean, (9) Madagascar, defined as the island of Madagascar and all other Indian Ocean islands in the vicinity, (10) India, defined as the area below 3000 m from NW Pakistan to the border with Myanmar, (11) Indonesia and Wallacea, defined as Myanmar, SE Asia, southern China, western Indonesia to Lydekker's Line; including the Lesser Sunda Islands but excluding Timor, Wetar and associated islands, which are Australasian in origin, and (12) Australasia, defined as everywhere east of Lydekker's Line but including Timor, Wetar and small nearby islands. Furthermore, there is evidence for the ancient (Triassic) presence of cycads in Antarctica (e.g. *Antarcticycas schopfii* Smoot, Taylor, et Delevoryas emend. Hermsen, T. N. Taylor, E. L. Taylor, et Stevenson, Smoot *et al*., 1985; Hermsen *et al*., 2006), and Greenland (e.g. *Anthrophyopsis crassinervis* Nathorst (Harris, 1926, 1932)). To reflect their increasingly recognized role in global plant biogeography (e.g. Estrella *et al*., 2019), we added Antarctica and Greenland to the geographic areas to consider the possibility that cycads colonized these continents. We choose these 14 areas to test biogeographic hypotheses and estimate implications from the breakup of ancient Mesozoic supercontinents, whereas Wallace's bioregions do not capture tectonic and geological changes.

Relying on the WCVP (Govaerts *et al*., 2021), the geographic distribution for all extant cycad species (column ‘geographic_area’ in the database) was categorized by coding the presence or absence of each species in each of the above‐defined areas. We also used data available in the literature (e.g. http://cycadlist.org/, Calonje *et al*., 2022). Occurrences of introduced species or marginally entering an area were not considered. Similarly, we coded the 60 fossil species of our dataset. However, fossil data provide evidence on the past geographic presence of taxa, but not about geographic absence due to the incompleteness of the fossil record. In BioGeoBEARS, but not yet in DECX, one can include this information by coding the geographic range of fossils with missing data ‘?’ instead of true absence (*useAmbiguities = TRUE* option). Coding presence in region A and unknown presence or absence in other regions mean that any geographic range including A will have tip likelihood of 1, and any geographic range excluding A will have a tip likelihood of 0. Fossil lineages thus represent a positive constraint on ancestral range estimates, which is conservative because it gives more weight to large ranges than to single‐area ranges. However, in most empirical analyses like in cycads, many species occur only in a single area, and a few are widespread. Hence, we performed the analyses with the positive constraint strategy, using ‘?’ for fossil species, and ran it again with the assumption that fossil ranges are the true ranges. We then compared the effects of these assumptions on ancestral range estimates. An overview of the global geographic distribution of extant and extinct Cycadales is presented in Fig. 1.

A time‐stratified geographic model was built using connectivity matrices that consider paleogeographic changes through time (Beeravolu & Condamine, 2016). Connectivity matrices specify constraints on area connectivity by coding 0 if any two areas are not connected or 1 if they are connected at a given period based on paleogeographic reconstructions (e.g. Blakey, 2008; Seton *et al*., 2012; Kocsis & Scotese, 2021). We created connectivity matrices to represent major changes in tectonic conditions that may have affected cycad distribution and to define biological plausibility of ranges over time. For instance, there are wide disjunctions between Australasia and North America such that no species is found on both. We did not add dispersal constraints because setting the values for dispersal rates between regions through time is subjective, and it has been shown that dispersal probability categories had minor effects on ancestral state estimates (Chacón & Renner, 2014). We assumed a dispersal matrix with equal rates between areas. The time‐slicing protocol introduced by Upchurch *et al*. (2002) is followed here. Four time slices were selected to construct the time‐stratified model and correspond to the major geological periods. The first covers the Carboniferous to the Late Triassic (358.9–201.3 Ma), corresponding to the assembly of Pangea (Kocsis & Scotese, 2021). The second ranges from the Early Jurassic to the Late Cretaceous (201.3–66 Ma), which represents the breakup of Pangea into Gondwana and Laurasia (Blakey, 2008; Seton *et al*., 2012). There are two time slices for the Cenozoic: one covers the Paleogene (66–23 Ma), and the other encompasses the Neogene to the present, corresponding to the tectonic plate motion to current positions. These time slices were designated because: (1) they are based on the major periods with important geological changes (Seton *et al*., 2012; Müller *et al*., 2016; Kocsis & Scotese, 2021); (2) subdivision of the Mesozoic into two time slices allows testing of biogeographic hypothesis (e.g. supercontinent breakups, Ezcurra & Agnolín, 2012); and (3) subdivision of the Cenozoic into Paleogene and Neogene with the latter witnessing the establishment of the present terrestrial biodiversity (Fine & Ree, 2006) and marked by a modern plate configuration (Kocsis & Scotese, 2021) and the establishment of the present climate regime during a long cooling process (Westerhold *et al*., 2020).

We used the most likely ancestral range estimates to count the biogeographic events such as dispersals ‘into’ and ‘out of’ a region as well as local extinctions (extirpations from a region). Biogeographic events can occur at nodes (cladogenesis) and along branches (anagenesis). We made a custom R script to retrieve the biogeographic events and calculate their timing across the phylogeny (see Data availability). Following previous studies (e.g. Antonelli *et al*., 2018; Meseguer & Condamine, 2020), anagenetic events were dated at middle points of branches and constrained by the time‐stratified geographic model to account for appearances and disappearances of regions and connectivities between them through time.

## Results

### Total‐evidence phylogeny of cycads

**Fig. 2:**
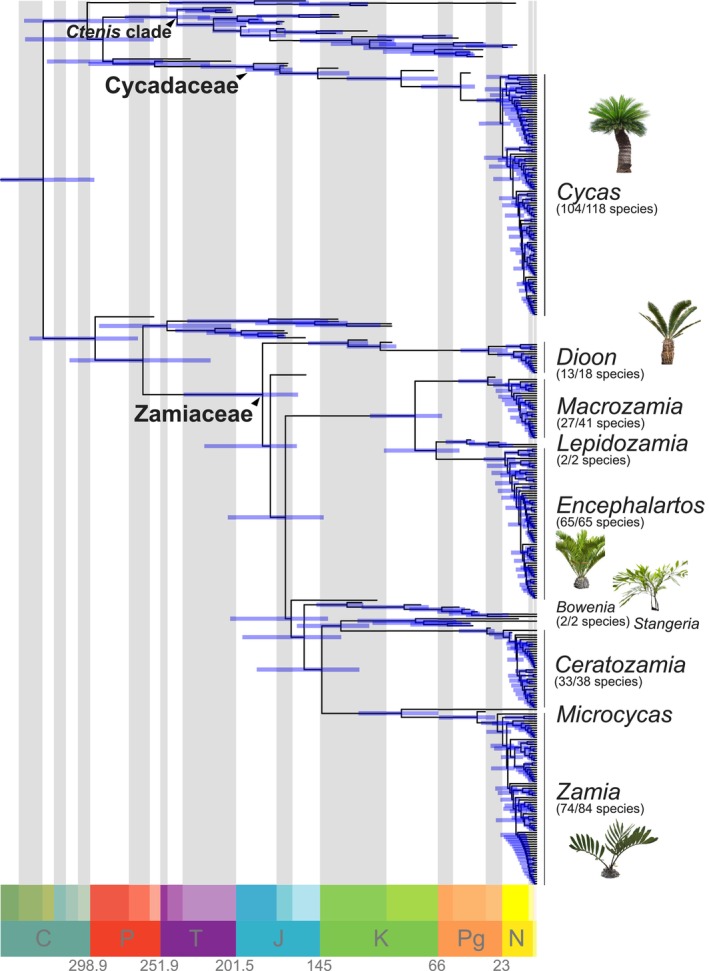
Bayesian total‐evidence dated phylogeny of Cycadales. This chronogram is the resulting consensus tree from the MrBayes analyses performed with the fossilized‐birth‐death model and an uncorrelated relaxed molecular clock. The tree includes 321 extant species and 60 extinct species with median divergence times along with 95% Highest Posterior Density (blue bars) for each node. C, Carboniferous; J, Jurassic; K, Cretaceous; N, Neogene; P, Permian; Pg, Paleogene; T, Triassic.

**Fig. 3:**
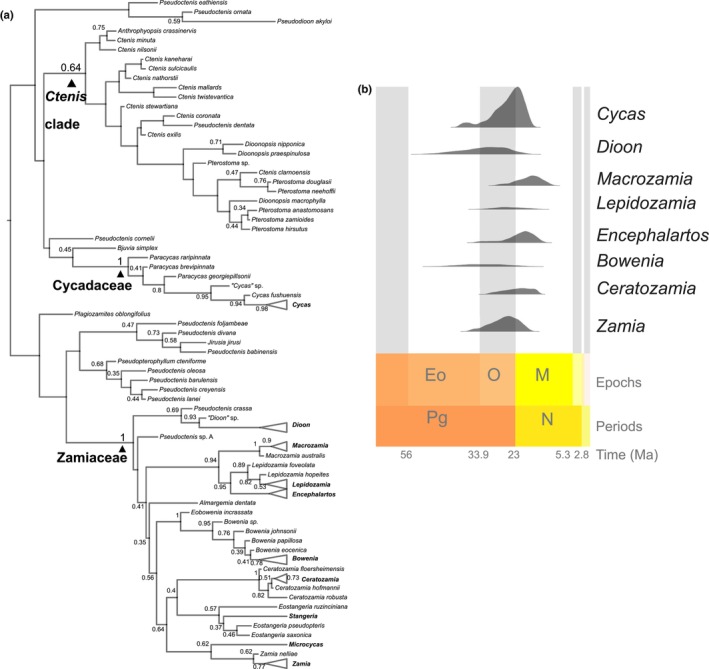
Ages of extant genera and fossil placements. (a) Phylogenetic relationships for extant (genera are collapsed) and extinct cycads with support values (Posterior Probability indicated when > 0.3). Node support for extant genera is only indicated when < 1. (b) Posterior distributions for the crown ages of the extant genera. Eo, Eocene; M, Miocene; N, Neogene; O, Oligocene; Pg, Paleogene. Ma, million years ago.

The ML phylogeny of cycads recovers generic relationships in agreement with the literature (Fig. S1) and is generally robust (UFBS ≥ 95, Fig. S2). However, many nodes within the extant genera remain unresolved. This topology served as backbone topological constraints to improve convergence of Bayesian inferences. After checking for convergence of the Bayesian total‐evidence analyses (ASDSF = 0.013; on average PSRF = 0.9999 and ESS = 623 for all parameters; *n* = 94), the consensus tree shows an extant‐genus topology in agreement with previous studies and that most cycad fossils are not closely related to the extant groups (Figs 2, S3, S4). Indeed, only five fossil taxa are strongly associated with Cycadaceae (i.e. are included in a clade with *Cycas* with PP = 1) including the extinct genus *Paracycas* and the two *Cycas* fossil species, and 18 are strongly associated with Zamiaceae (Fig. 3). Many relationships based on morphological comparative analyses are retrieved, that is, *Ceratozamia hoffmannii* Ettinghausen and *Ceratozamia floersheimensis* (Engelhardt) Kvaček form a clade with *Ceratozamia* (PP = 1), *Macrozamia australis* Carpenter is sister to *Macrozamia* (PP = 1), and *Lepidozamia hopeites* (Cookson) L. Johnson and *Lepidozamia foveolata* are sister to *Lepidozamia* (PP = 0.82 and 0.89, respectively). Species of the genus *Eostangeria* Barthel are retrieved in a clade with extant *Stangeria*, though with lower levels of support (PP = 0.57; Fig. S5). Likewise, extinct species of the genus *Bowenia* are retrieved in a clade with extant *Bowenia* with high support (PP = 0.95), and sister to genus *Eobowenia* with maximal support (PP = 1). The relationships between fossil taxa are less strongly supported than the relationships between fossil and extant taxa. There are 27 fossil species along the stem Cycadaceae and 10 fossil species along the stem Zamiaceae; most of them have low node support (PP < 0.5). Nonetheless, clades' PP > 0.5 are retrieved for several groups of fossil taxa, indicating that even a small morphological matrix can contain some phylogenetic informativeness (Fig. 3).

The fossil leaves *Dioonopsis praespinulosa* (Hollick) Erdei, Manchester et Kvaček and *Dioonopsis macrophylla* (Potbury) Erdei, Manchester et Kvaček, which have been previously associated with extant *Dioon* and even used as calibrations for molecular dating (Nagalingum *et al*., 2011; Condamine *et al*., 2015; Gutiérrez‐Ortega *et al*., 2018), are not related to *Dioon* nor Zamiaceae, but are nested in an extinct clade including *Ctenis* species along the stem branch of Cycadaceae (Fig. S5).

Our analyses do not support the monophyly of any fossil cycad genus (Fig. 3). The genus *Pseudoctenis* Seward, defined as having broadly attached multiveined leaflets like *Ctenis* but lacking anastomoses, is wildly polyphyletic. It includes taxa related to the Zamiaceae (i.e. *Pseudoctenis* species A from the Late Jurassic of France and *Pseudoctenis crassa* S.Archang. & Baldoni from the Early Cretaceous of Argentina (Archangelsky & Baldoni, 1972)), taxa nested among species assigned to *Ctenis* (*Pseudoctenis dentata* S.Archang. & Baldoni from the Early Cretaceous of Argentina), as well as two clades including respectively *Pseudoctenis* species from the Jurassic as well as the genus *Pseudopterophyllum* Florin, and *Pseudoctenis* species from the Cretaceous and the genus *Jirusia* Bayer. A relationship between the Early Cretaceous *Pseudoctenis ornata* A.Archang., R.Andreis, S.Archang. & A.Artabe, and the Miocene *Pseudodioon akioly* Erdei, Agkun & Barone Lumaga is also retrieved (0.59). Interestingly, the type species of the genus (*Pseudoctenis eathiensis* (Richards) Seward) does not receive appreciable support for any placement.

The phylogenetic analysis finds some evidence (although not strong) of a long‐lasting lineage of cycads that did not leave any extant representatives. This clade includes the mostly Mesozoic genus *Cteni*s together with the Cenozoic genera *Pterostoma* Hill and *Dioonopsis* Horiuchi & Kimura (supported with PP = 0.64). Such a lineage was postulated on comparative grounds because of the presence of peculiar H‐anastomoses in the venation of these leaves, a character absent in all extant cycads (Erdei & Manchester, 2015). However, not all members of the lineage retrieved here possess anastomoses (i.e. *Pseudoctenis dentata*).

### Total‐evidence dating of cycads

Using the FBD model, the Bayesian dating analyses estimate the divergence between Zamiaceae and Cycadaceae *c*. 330 Ma at the boundary between the Early and Late Carboniferous (Fig. 2; 95% HPD = 296.2–358.9 Ma). These results place the split at an older date than the previous node‐dating analyses (Table 1). The origin of the crown‐group Zamiaceae is estimated at 183.5 Ma between the Late Triassic and the Late Jurassic (95% HPD = 159.8–236.3 Ma). *Bowenia* is inferred to have split from its sister clade *c*. 155.6 Ma between the Early Jurassic and the Early Cretaceous (95% HPD = 130.9–197 Ma). Zaminae (genera *Ceratozamia*, *Stangeria*, *Zamia*, and *Microcycas*) appears to have an older crown age than Encephalartinae (genera *Encephalartos*, *Lepidozamia*, *Macrozamia*), with the former clade originating at 143.7 Ma between the Jurassic and Early Cretaceous (95% HPD = 118.8–187.3 Ma), and the latter originating at 81.3 Ma between the Early Cretaceous and the Paleocene (95% HPD = 63.2–111.5 Ma). A Cretaceous age is inferred for the divergence between *Microcycas* and *Zamia* (95% HPD = 65.7–119.3 Ma).

**Table 1:** Ages for the main cycad clades retrieved in our analysis and compared with ages reported from the node‐calibration analysis of Condamine *et al.* (2015).

Cycad clades	Ages (Ma) from Condamine *et al*. (2015)			Ages (Ma) from the total‐evidence dating		
	Median	Min	Max	Median	Min	Max
Cycadales	274.5	235.0	332.4	330.4	296.2	358.9
Zamiaceae	156.1	107.0	207.9	183.5	159.8	236.3
Zaminae	107.8	74.3	147.7	143.7	118.8	187.3
Encephalartinae	56.2	39.3	82.1	81.3	63.2	111.5
*Ceratozamia‐Stangeria*	84.9	55.0	118.9	131.0	112.1	160.4
*Encephalartos‐Lepidozamia*	39.1	33.9	55.0	67.2	51.7	102.1
*Zamia‐Microcycas*	57.0	34.2	84.5	90.5	65.5	119.3
*Cycas*	17.6	10.1	29.1	24.4	18.1	40
*Bowenia*	5.5	0.8	14.6	32.8	18.7	47.2
*Dioon*	15.4	7.5	24.6	32.1	19.4	50.9
*Ceratozamia*	19.2	9.5	33.2	22.2	16.5	31.6
*Encephalartos*	10.5	6.2	16.3	21.1	14.2	35.45
*Macrozamia*	9.1	5.1	15.3	18.6	11.7	28.8
*Lepidozamia*	10.9	3.2	23.1	25.3	15.7	34.3
*Zamia*	14.6	9	22.1	26.3	18.4	37.2

For the latter study, only ages from the analysis with the full fossil dataset and the birth‐death tree prior are reported. Min and max ages represent the upper and lower boundary of the 95% Highest Posterior Density. Ma, million years ago.

Genus crown ages inferred in our analyses are summarized in Table 2. Even though we do retrieve a relatively young origin of extant species diversity (Fig. 3), the hypothesis of a synchronous radiation of the extant genera is weakened in our analysis. Based on a sample of 100 random trees from the posterior, a repeated measurement ANOVA retrieves significant differences between the crown ages of the genera (*P* ≤ 0.0001). *Post hoc* testing finds significant differences between the mean ages of most genera (Table 2).

**Table 2:** Ancestral ranges for main cycad clades as estimated with the biogeographic analyses under the Dispersal–Extinction–Cladogenesis model (14 areas and four time slices) applied to the phylogeny including extant species only or extant and extinct species.

Cycad clades	Median age (Ma)	Ancestral ranges estimated with extant and extinct cycads				Ancestral ranges estimated with extant cycads only			
		Best	Second	Third	Interpretation	Best	Second	Third	Interpretation
Cycadales	330.4	WP + EP	WP	WP + EP + GR	Laurasia	WP + EP + EN + AF + AN	WP + EN + AF + AU + AN	WP + EP + EN + AF	Pangea
Zamiaceae	183.5	WP + EN + CA + SA + GR	WP + EN + WI + SA + GR	WP + EN + AF + GR + AN	Pangea	CA + AF + IN+AU + AN	CA + WI + AF + AU + AN	EN + CA + AF + AU + AN	Gondwana
Zaminae	143.7	WP + EN + CA + GR	WP + EN + WI + GR	WP + EN + CA + WI + GR	Laurasia	CA + AF	CA + WI + AF	CA + AF + AN	Western Gondwana
Encephalartinae	81.3	EN + WI + SA + AU + AN	CA + WI + SA + AU + AN	WI + SA + AU + AN	Western Gondwana	AU	–	–	Australasia
*Ceratozamia‐Stangeria*	131.0	WP	WP + GR	WP + EN + GR	Laurasia	CA + AF	–	–	Western Gondwana
*Encephalartos‐Lepidozamia*	67.2	EN + WI + SA + AU + AN	CA + WI + SA + AU + AN	–	Western Gondwana	AU	–	–	Australasia
*Zamia‐Microcycas*	90.5	CA + WI	CA	WI	Central America	CA + WI	CA	WI	Central America
*Cycas*	24.4	EP + WA	WA	–	Eastern Asia	WA	EP + WA	–	Eastern Asia
*Bowenia*	32.8	AU	–	–	Australasia	AU	–	–	Australasia
*Dioon*	32.1	CA	–	–	Central America	CA	–	–	Central America
*Ceratozamia*	22.2	WP + EP + WN + CA	–	–	Holarctic	CA	–	–	Central America
*Encephalartos*	21.1	EP + AF + WA + AU	EP + AF + WA	–	Paleotropics	EP + AF + WA + AU	EP + AF + WA	–	Paleotropics
*Macrozamia*	18.6	AU	–	–	Australasia	AU	–	–	Australasia
*Lepidozamia*	25.3	AU	–	–	Australasia	AU	–	–	Australasia
*Zamia*	26.3	CA	–	–	Central America	CA	CA + WI	–	Central America

For each clade, the most likely ancestral range (Best) as well as the second and third most likely range are reported. A range can be composed of several areas (denoted with +). Area abbreviations defined as follows: WP, West Palearctic; EP, East Palearctic; WN, West Nearctic; EN, East Nearctic; CA, Central America; WI, Caribbean Islands; SA, South America; AF, Africa; IN, India; WA, Southeast Asia; AU, Australasia; GR, Greenland; AN, Antarctica. The Interpretation column translates the estimated ancestral ranges into major biogeographical and geological entities. Ma, million years ago.

### Historical biogeography and latitudinal distribution through time

**Fig. 4:**
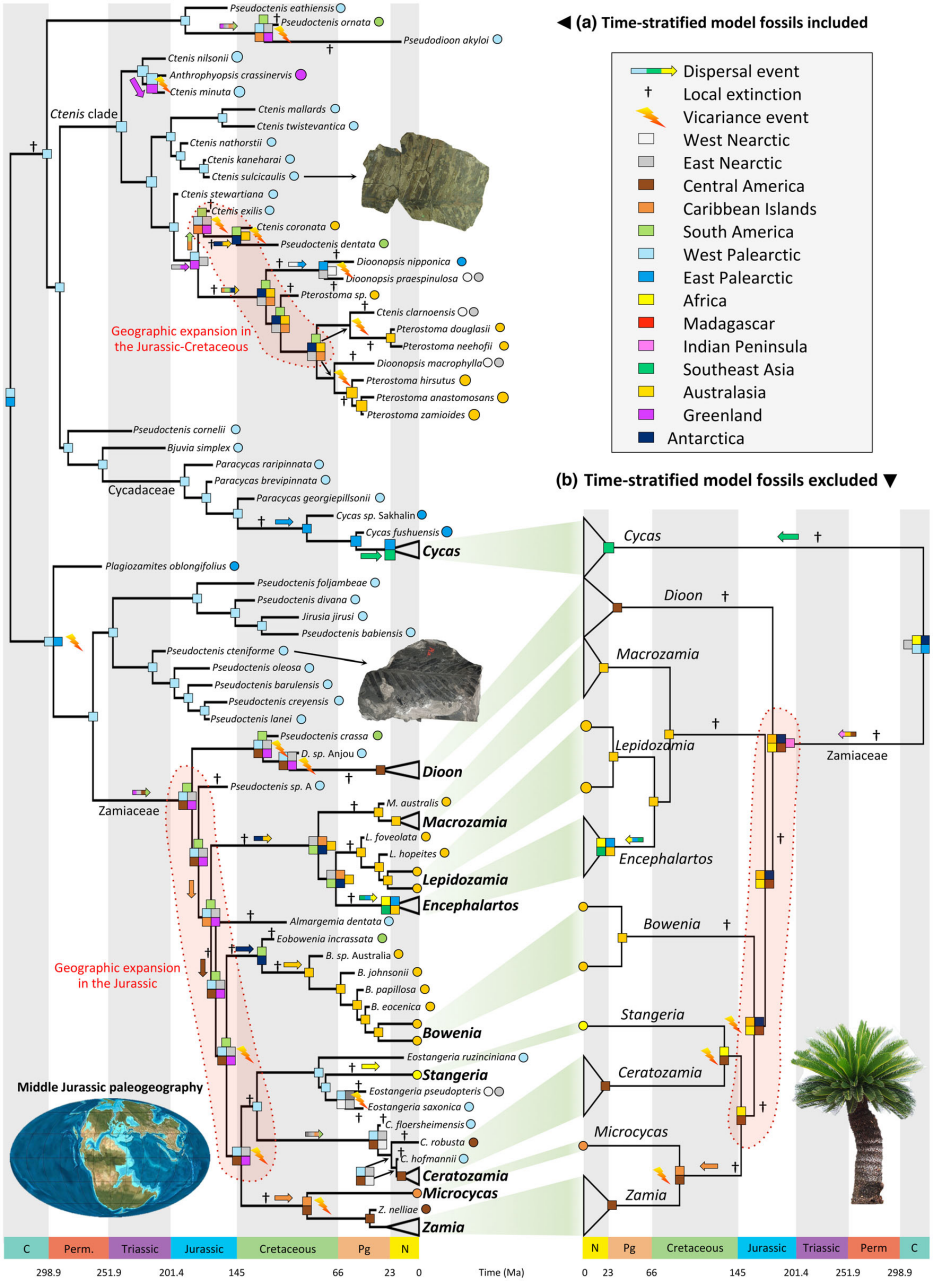
Historical biogeography of cycads. Estimates of ancestral areas were performed with a time‐stratified model in Dispersal–Extinction–Cladogenesis (DEC) with fossils included (a) and with fossils excluded (b). The extant genera have been collapsed to focus on deep‐time biogeography (for details within each genus, see Supporting Information Fig. S6 when fossils are included and Fig. S7 when fossils are excluded). The bottom‐right corner legend indicates colored areas used in this study corresponding to colored squares for each node, representing inferred ancestral area(s) with the DEC model, and colored circles for fossil species representing known distributions (except for extant genera with one or two species only). The red‐highlighted shades show the cycad expansion into Gondwana during the Jurassic and Cretaceous. The bottom‐left corner map represents the global paleogeography in the Jurassic (180 million years ago (Ma)). Paleomap used with permission © 2020 Colorado Plateau Geosystems Inc. Arrows indicate fossil species illustrated. Pictures from Mario Coiro. C, Carboniferous; N, Neogene; Perm, Permian; Pg, Paleogene.

When including both extant and extinct cycads (Fig. S6), the DEC analyses estimated a most likely ancestral origin for Cycadales in Western and Eastern Palearctic (Fig. 4, relative probability = 0.192). The second‐best inference included only Western Palearctic (relative probability = 0.166), and the third best encompassed Western‐Eastern Palearctic and Greenland (relative probability = 0.140). Only geographic areas of Laurasia are recovered as part of the estimated ancestral ranges for the cycad origin (cumulative relative probability = 0.788). When including only extant cycads (Fig. S7), the DEC analyses found a widely Pangean origin with the most likely range comprising Western–Eastern Palearctic, Eastern Nearctic, Africa, and Antarctica. Without fossils, there was higher uncertainty at the root ancestral range. There were 22 and 10 ‘equally likely’ ancestral ranges (i.e. ranges having a log‐likelihood lower than 2 units compared with the most likely range) for the root when excluding and including fossils, respectively. Generally, the discrepancies of biogeographic inferences between the two analyses are found in the deep nodes (e.g. crowns of Zamiaceae, Zaminae, and Encephalartinae; Fig. 4; Table 2). These results were robust when coding fossil geographic ranges with missing data instead of true absence (Figs S8, S9) and with uncertainties in fossil placements and age estimates (Fig. S10).

**Fig. 5:**
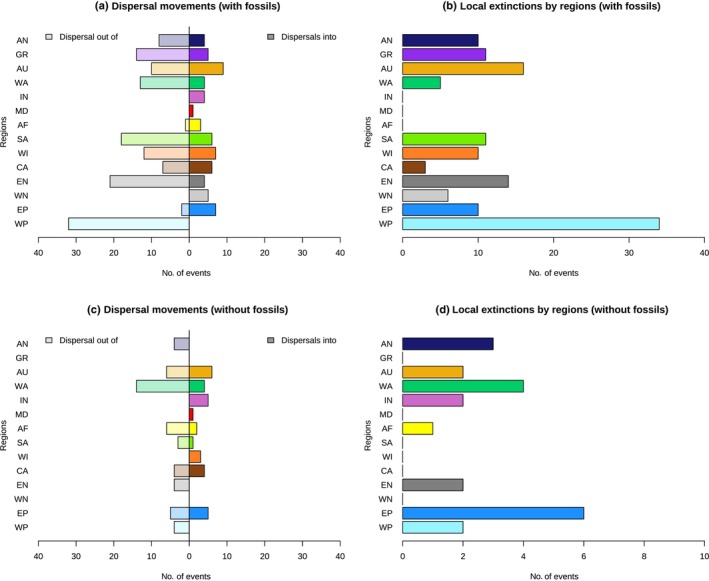
Biogeographic processes explaining the global distribution pattern of cycads. The number of dispersal events into a region and out of a region as well as the number of local extinctions (extirpations) are compared between analyses including fossils (a, b) and analyses excluding fossils (c, d). Area names: WP, West Palearctic; EP, East Palearctic; WN, West Nearctic; EN, East Nearctic; CA, Central America; WI, Caribbean Islands; SA, South America; AF, Africa; IN, India; WA, Southeast Asia; AU, Australasia; GR, Greenland; AN, Antarctica.

Using ancestral estimates, we extracted biogeographic processes (dispersals, local extinctions (= extirpations), and vicariance) explaining the geographic range evolution in cycads. Since determining which area the dispersal event came from with an ancestral range including two or more areas is challenging, we counted one dispersal event per area of the ancestral range that likely leads to an overestimation of dispersal events. Comparing biogeographic processes between the phylogenies including or excluding fossils, we found differences in the extent and number of events (Fig. 5). Dispersal events were separated into two categories: dispersal into a region and dispersal out of a region (Fig. 5a,c). We estimated 25 dispersals into high‐latitude regions with fossils included, but only 5 when fossils are excluded. We further found 40 dispersals into low‐latitude regions compared with 26 when fossils are excluded. Moreover, we estimated 77 dispersals out of high‐latitude regions with fossils included, while only 17 when fossils are excluded. We also recovered 61 dispersals out of low‐latitude regions compared with 33 when fossils are excluded. Despite these discrepancies, there are also similarities such as the role of the Indomalayan region as a source of dispersal as well as Central and South America to a lesser extent, or East Palearctic and Australasia as sinks of diversity.

The biogeographic analyses revealed numerous vicariance events (Fig. 4), with 32 events inferred with fossils and 20 events without fossils. Local extinctions, or extirpations (Figs 5b,d, S11), are fifth as often inferred when fossils are included (130) than when they are excluded (22). Among extirpations, we found 85 high‐latitude and 45 low‐latitude extirpations when including fossils, while there are only 13 and 9 when fossils are excluded.

**Fig. 6:**
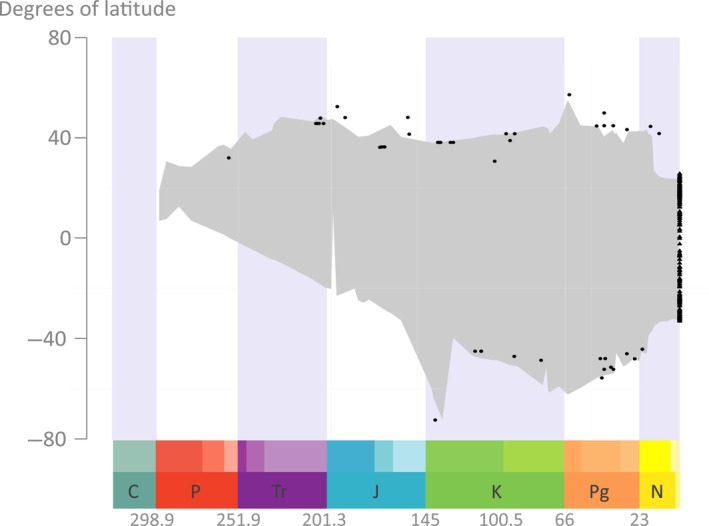
Reconstruction of the latitudinal span of cycads during geological times. This estimate has been obtained using the method of Silvestro *et al*. (2018b) as implemented in Zhang *et al*. (2022). Gray polygon represents the reconstructed span, black dots represent the paleolatitude and age of fossil tips, while black triangles show the latitude of extant species. C, Carboniferous; J, Jurassic; K, Cretaceous; N, Neogene; P, Permian; Pg, Paleogene; Tr, Triassic.

The analysis of latitudinal distribution indicates a mid‐to‐low northern latitude origin of the Cycadales (Fig. 6). A similar latitudinal range is maintained until the Late Jurassic, when the group spreads to high latitudes in the Southern Hemisphere. During the Cretaceous and Paleogene, the group persists at mid‐to‐high latitudes in both Hemispheres. A contraction between the end of the Paleogene (Oligocene) and the mid‐Miocene leads to the current subtropical to tropical distribution.

## Discussion

Our results show that the integration of fossils as tips in a total‐evidence phylogeny of cycads provides a more complete view of their macroevolutionary history. Here, we discuss the consequence of our results on cycad phylogeny and how this new phylogenetic hypothesis informs us on the biogeographic history processes explaining global cycad biodiversity.

### Phylogeny of cycads

Our study shows the feasibility and advantages of the total‐evidence approach for cycads, and it allows us to reach insights that would be impossible using other approaches. Our analyses provide new insights into long‐standing issues with the cycad phylogeny. Most Triassic and Early to Middle Jurassic cycad leaves are not particularly closely related to extant cycad families, contrary to previous suggestions based on morphology (Hermsen *et al*., 2006; Martínez *et al*., 2012) or morphology + molecules (Coiro & Pott, 2017). Previous studies may have been swayed by the coding of polyphyletic genera as single taxa, and the inclusion of only a few characters relating to leaf morphology and anatomy. The polyphyletic nature of fossil leaf genera in Cycadales is revealed in our analyses, particularly for the genus *Pseudoctenis* (Pott *et al*., 2007). This suggests that the use of generic‐level analyses as a proxy for species‐level dynamics (Roy *et al*., 1996) should be viewed with caution. Coding of taxa at the level of single species and the implementation of the FBD prior could represent a more promising avenue in groups with a poorly understood fossil record and without many whole‐plant reconstructions, a widespread situation in land plants.

Our results validate some previous suggestions of relatedness between fossil taxa and extant genera (Hill, 1978; Carpenter, 1991; Kvaček, 2002; Su *et al*., 2014; Erdei *et al*., 2018; Hill *et al*., 2019a). Previously puzzling forms, such as *Eostangeria* (Barthel, 1976; Kvaček & Manchester, 1999; Uzunova *et al*., 2001), appear to represent transitional forms between the *Zamia*‐*Microcycas* clade and the extremely derived genus *Stangeria*, combining the epidermal anatomy of the former with the unique macromorphology of the latter (i.e. leaflets with pinnate venation). This contrasts with the idea of *Eostangeria* as a separate lineage in the Zamiaceae that converged with *Stangeria*. The tree also confirms the relationship between the Mesozoic genus *Paracycas* Harris and extant *Cycas*. The genus *Eobowenia* is confirmed as sister to extant *Bowenia*, strengthening inferences on its understory habit based on comparison with other fossil and extant species of this lineage (Hill *et al*., 2019b).

Another important result is the emergence of the hypothesis of an entirely extinct clade spanning the Triassic to the Miocene, including both common Mesozoic forms assigned to *Ctenis* and controversial Cenozoic fossils such as *Pterostoma* (Florin, 1933; Harris, 1964). The morphology of the leaves in this clade differs from that of the two extant families mostly in the presence of H or N anastomoses. This clade includes *Dioonopsis* validating results based on comparative investigations (Barone Lumaga *et al*., 2015; Erdei & Manchester, 2015), and weighing strongly against its use as a calibration for the age of *Dioon* (e.g. Li *et al*., 2019).

The phylogenetic distance between most fossil cycads and the extant clades argues against the use of extant cycadalean ecophysiology to infer past preferences of fossil cycads (McElwain, 1998), especially for members of the *Ctenis* clade. Indeed, the only *Ctenis* species for which we have a good paleoecological understanding (Wing *et al*., 1993) occupied a fern‐dominated open habitat on a peaty substrate, unlike any extant cycad. More studies are needed to better understand the ecology of fossil cycads and provide clues into the causes of their extinction.

### Origin and early historical biogeography of cycads

Our dating analyses first show a more ancient Paleozoic age for the origin of Cycadales (Carboniferous vs Permian or even Triassic in molecular‐only studies; Nagalingum *et al*., 2011; Salas‐Leiva *et al*., 2013; Condamine *et al*., 2015). Furthermore, our results indicate a much wider range in the ages of crown‐group genera (Fig. 3b). Despite clear limits in our study (to be described later), this new phylogenetic framework allows the estimation of the historical biogeography and latitudinal range evolution of Cycadales. Nonetheless, we found that the ancestral state estimates were robust when coding fossil geographic ranges with missing data instead of true absence and when considering uncertainties in fossil placements and divergence times.

To our knowledge, only one study attempted to reconstruct the geographic origin of cycads with phylogenetic approaches, showing an origin in Australia, China, and Mexico (Salas‐Leiva *et al*., 2013). However, such a geographic range is unlikely given the nonadjacency of the three areas, an issue probably due to the lack of a time‐stratified geographic model. Here, by including Antarctica and Greenland with now‐vanished continental connections, we relied on more accurate information from a paleogeographic perspective. We also compared the effect of including the fossils into the ancestral estimates. We show important discrepancies between the analyses including or not the fossils as tips, particularly in deep nodes. This could be expected given the long branches subtending radiations of extant genera bearing little phylogenetic information in deep times, supporting the view of Crisp *et al*. (2011) that fossil lineages are crucial to study historical biogeography. However, treating fossil distributions as unknown did not impact the analysis dramatically. This could be due to the high endemism of cycad species (only 11 species out of 381 have more than one area for their range), thus limiting the possibility of unobserved presence of widespread fossil species.

Cycadales likely originated in the northern part of Pangea (Laurasia). This result agrees with the most ancient cycad fossil lineage, *Crossozamia* Pomel, described from the Permian of China (Gao & Thomas, 1989). The Carboniferous was a time of active mountain‐building as the supercontinent Pangea coalesced (von Raumer *et al*., 2003). The southern continents were united as Gondwana, which collided with the West Palearctic and East Nearctic. This collision resulted in the Hercynian orogeny in Palearctic, and the Alleghenian orogeny in Nearctic (von Raumer *et al*., 2003). These mountain ranges could have limited cycad distribution in northern Pangea. Contemporaneously, much of present eastern Eurasian plate welded itself to the West Palearctic along the Ural Mountains, which makes plausible an eastern Laurasian origin for cycads. During the Carboniferous, global climate progressively cooled and dried, eventually culminating with an extinction period known as the Carboniferous rainforest collapse that drastically affected terrestrial biodiversity in Laurasia (Sahney *et al*., 2010) of which cycads survived. Cycads remained in Laurasia (including Greenland), potentially constrained by the Central Pangean Mountains in the Permian. By the Middle Triassic, the Central Pangean mountains had been substantially reduced in size, and by the earliest Jurassic *c*. 200 Ma the Pangean range in West Palearctic was reduced to upland areas surrounded by marine basins (Scotese & Schettino, 2017). In the Early Jurassic, our inferences suggest Zamiaceae and the *Ctenis* clade independently colonized Gondwana through East Nearctic and Central America or Caribbean going to South America.

### The role of Antarctica and Greenland

From the Jurassic until the end‐Cretaceous, cycads expanded to all continents through active dispersals into low‐latitude regions of Gondwana. Four ancient independent colonizations of Antarctica were inferred between the Late Jurassic and Early Cretaceous (two in the *Ctenis* clade between 174 and 147 Ma and 179 and 123 Ma; *Eobowenia* and *Bowenia* between 155 and 130 Ma; Encephalartinae between 170 and 81 Ma). In line with the dense fossil record of Patagonia (Artabe & Stevenson, 1999; Cúneo *et al*., 2010), our analyses indicate West Gondwana is a biogeographic crossroad for cycads with numerous (18) dispersals out of South America. At that time, South America was connected to Antarctica, itself connected to Australia (Blakey, 2008; Seton *et al*., 2012; Kocsis & Scotese, 2021), thus creating a large land bridge for terrestrial biodiversity.

The case of Encephalartinae illustrates well the impact of fossils and how they help clarify their origins. The African genus *Encephalartos* is sister to *Lepidozamia* endemic to Australia, separated at the K/Pg boundary (67 Ma), and both are sister to *Macrozamia*, which is also restricted to Australia and diverged in the Late Cretaceous (81 Ma). Both *Lepidozamia* and *Macrozamia* have fossil taxa distributed in Australia, and the Patagonian *Austrozamia stockeyi* Wilf, D.Stevenson et Cuneo is thought to be related to the *Encephalartos*‐*Lepidozamia* clade (Wilf *et al*., 2016). However, no fossil taxon is known to be closely related to *Encephalartos*. Our analyses suggest a widespread ancestor in Gondwana (including Antarctica) for Encephalartinae and the common ancestor of *Encephalartos* and *Lepidozamia*. However, this wide range does not include Africa, which was not connected to other Gondwanan continents in the Late Cretaceous. We estimated the colonization of Africa along the stem of *Encephalartos* likely started from Australia and extended northward to Southeast Asia and passing through the East Palearctic, followed by numerous Paleogene extirpations. If correct, we can expect to discover fossil taxa possibly related to *Encephalartos* in Cenozoic deposits of Eurasia. However, the presence of fossils with potential affinities with Encephalartinae in South America could also indicate a Western Gondwanan route, with dispersal to Africa via Antarctica supported by the South African origin of *Encephalartos* (Mankga *et al*., 2020a). The lack of stomatal morphology in these South American fossils unfortunately does not allow testing of this hypothesis. Interestingly, the analyses without fossils recover the same range for *Encephalartos*, but an Australasian origin for Encephalartinae.

Likewise, Greenland was key in cycad biogeography. We recovered five independent colonizations from the Late Triassic (two in the *Ctenis* clade) to the Early Jurassic (in Zamiaceae). Greenland was particularly prominent in ancestral range estimates for Zamiaceae and acted as biogeographic crossroads for all lineages of the backbone (14 dispersals out of Greenland). Our study highlights the role of Antarctica and Greenland in biogeographic estimates as proposed previously (de la Estrella *et al*., 2019). Indeed, Antarctica and Greenland were, separately, sources of common ancestors until the Paleocene–Eocene transition, after which the local climate became unfavorable for plant growth (Pross *et al*., 2012; Suan *et al*., 2017; Klages *et al*., 2020). Importantly, we estimated that nine vicariance events out of a total of 31 involve Antarctica or Greenland, when added together, with subsequent disjointed descending lineages. The recognition of the role of Antarctica and Greenland in historical biogeography has rarely been implemented in macroevolutionary analyses. We argue that the incorporation of fossils allows recovering the role of these continents, which is otherwise challenging without them. However, our analyses excluding fossils did recover the role of Antarctica in the early nodes of Zamiaceae, but not of Greenland.

Our results including or excluding the fossils indicate similar geographic origins for the crown of extant genera, except for *Cycas* and *Ceratozamia*. We find different ancestral ranges because of the inclusion of one East Palearctic and two West Palearctic fossils, respectively. For *Ceratozamia*, the discrepancy is stronger because *C. hofmannii* (early Miocene of Austria) is found within the extant genus, unlike a stem lineage for *C. floersheimensis* (early Oligocene of Germany). Hence, we estimated a broad Laurasian range from the early Oligocene to early Miocene followed by geographic extirpations in the Neogene. Although the impact of fossils is less obvious toward the present, extinct taxa bear direct evidence of the presence of related extant representatives that can occur in different regions. Including them can alter the inference of evolutionary processes explaining their current distribution pattern.

Within the most species‐rich cycad genera, *Cycas* and *Zamia* show a dynamic biogeographic history with multiple area colonizations by range expansions and several vicariance events (11 for *Cycas* and 6 for *Zamia*). *Zamia* colonized the Caribbean Islands during the second emersion of the Aves Ridge 16 Ma (Garrocq *et al*., 2021). Our analyses underestimate the number of allopatric events because of the broad‐scale analyses with continents as biogeographic units. Finer‐scale studies have already unveiled evolutionary processes explaining extant species distribution within genera and showing the role of allopatry (Calonje *et al*., 2019; Mankga *et al*., 2020b; Habib *et al*., 2022).

### Evolution of the latitudinal gradient of cycad biodiversity

We complemented historical biogeography with ancestral latitude estimates. Although the origin of cycads at high latitude might be due to the poor record in tropical and equatorial latitudes, our analysis clearly shows that cycads were heavily impacted at the end of the Paleogene and in the mid‐Miocene (40 high‐latitude extinction events vs 22 low‐latitude extinction events, Fig. S10). This period corresponds with the extinction of the *Ctenis* clade, as well as the disappearance of many relatives of extant genera from Europe and North America. Interestingly, many Cenozoic species of the *Ctenis* clade are distributed at high latitudes (*Pterostoma* in Australia and New Zealand, *Dioonopsis* in Alaska). This pattern is reminiscent of the high‐latitude refugium hypothesis advanced for many fossil and extant groups (Bomfleur *et al*., 2018), including ferns that cooccurred with *Ctenis* in the past (Wing *et al*., 1993). Testing such a hypothesis on mechanistic grounds could represent an interesting avenue for further investigations.

Elevated extinction of gymnosperms during the Cenozoic has been proposed as a partial explanation for the imbalance in species richness between gymnosperms and angiosperms (Crisp & Cook, 2011; Condamine *et al*., 2020), and are retrieved in analyses of both phylogenies (May *et al*., 2016) and the fossil record (Crepet & Niklas, 2009). Whether such an elevated extinction was driven by one or more events remains hard to infer, but phylogeny‐based diversification models unveil an increasing extinction rate in cycads, with diversity decline starting 125 Ma (Mazet *et al*., 2022). The succession of many cooling events, in the Maastrichtian (Linnert *et al*., 2014), at the Eocene–Oligocene transition (Houben *et al*., 2012), the Oligocene–Miocene transition (Beddow *et al*., 2015), and the mid‐Miocene transition, leading to an Icehouse Earth, could have led high‐latitude cycads to extinction, and shifted the distribution of the group to more subtropical and tropical latitudes (Meseguer & Condamine, 2020). The maximum latitudinal expansion of the group broadly corresponds to a period of Greenhouse Earth with terrestrial ecosystems extending to Antarctica and Greenland (Suan *et al*., 2017; Klages *et al*., 2020).

Such periods of elevated extinction offer a better explanation for the patterns of diversification of the extant genera. Even if the origins of the crown groups of the extant genera appear not to be entirely synchronous, the ages retrieved in the tree are still relatively young. If this would go against the idea of a global ‘trigger’ for the radiation of the genera, it would be compatible with a period of elevated turnover. This would indicate that cycad evolution has been mainly influenced by abiotic factors (Barnosky, 2001; Benton, 2009). However, the influence of biotic factors cannot be fully excluded: The distribution of the members of the *Ctenis* clade in high‐latitude environments might be a consequence of competitive exclusion from lower‐latitude environments, which might have predisposed the clade to eradication by Neogene climatic change.

### Conclusion

Although our dated phylogeny includes a fraction of the known fossil cycad diversity, this study represents a step forward in our understanding of the global cycad biodiversity. Cycads originated in the Carboniferous on the Laurasian landmass and colonized Gondwana in the Jurassic. We revealed the crucial role of Antarctica and Greenland as biogeographic crossroads and found numerous vicariance events in the deep and recent past. Cycad latitudinal range increased in the Jurassic but retreated toward subtropical latitudes in the Neogene due to important high‐latitude extirpations. However, we remain cautious about our biogeographic inferences. For instance, we were unable to incorporate taxa like *Antarcticycas* (Triassic of Antarctica) or *Crossozamia* (Permian of China) because of the lack of preserved leaf traits. We think that including more fossil cycad taxa can alter phylogenetic relationships, which in turn can affect divergence times and biogeographic inferences. Further studies are needed to incorporate new cycad taxa to deepen our understanding of cycad origin and range evolution. This species‐based analysis represents a starting point for building a more species‐rich and character‐rich matrix that avoids the pitfall of previous analyses, namely the use of polyphyletic operational taxonomic units. Such an effort would require the reinvestigation of the fossil record of other cycad organs, and the establishment of more whole‐plant reconstructions for this group.

## Competing interests

None declared.

## Author contributions

MC and FLC conceived the project. MC, RA, LJS and FLC extracted data for the analyses. MC, RA, NM, LJS and FLC analyzed data. MC and FLC wrote the first draft of the manuscript and prepared figures. All authors edited the manuscript.

## Supporting information


**Fig. S1** Maximum‐likelihood phylogeny of Cycadales.
**Fig. S2** Node support for phylogenetic analyses of Cycadales.
**Fig. S3** Bayesian chronogram from the total‐evidence dating analysis including extant and extinct taxa.
**Fig. S4** Bayesian chronogram from the total‐evidence dating analysis excluding the fossil taxa.
**Fig. S5** Phylogenetic placements of fossil cycads illustrated using RoguePlots.
**Fig. S6** Estimate of the historical biogeography for Cycadales with extant and extinct species.
**Fig. S7** Estimate of the historical biogeography for Cycadales with extant species only.
**Fig. S8** Estimate of the historical biogeography for Cycadales using BioGeoBEARS (DEC model) with extant and extinct species by coding fossil geographic ranges with missing data instead of true absences (option *useAmbiguities = TRUE*).
**Fig. S9** Estimate of the historical biogeography for Cycadales using BioGeoBEARS (DEC model) with extant and extinct species by coding fossil geographic ranges as true absences (option *useAmbiguities = FALSE*).
**Fig. S10** Estimate of the historical biogeography for Cycadales using BioGeoBEARS (DEC model) with extant and extinct species by taking into account the uncertainties in fossil placements and divergence times, and coding fossil geographic ranges with missing data (option *useAmbiguities = TRUE*).
**Fig. S11** Number of local extinctions (extirpations) per time bin (Cenozoic vs Mesozoic and Paleozoic) compared between analyses excluding fossils and analyses including fossils.
**Methods S1** Examination of fossil specimens.
**Notes S1** Morphological characters used in the total‐evidence dating analyses.
**Table S1** Specimens re‐examined during the coding of the matrix in this study.
**Table S2** Fossil species used in this study, and references for ages and morphology.
Please note: Wiley is not responsible for the content or functionality of any Supporting Information supplied by the authors. Any queries (other than missing material) should be directed to the *New Phytologist* Central Office.

## Data Availability

The data that support the findings of this study and code used in this study are openly available in FigShare at doi: 10.6084/m9.figshare.21399576.v1.
