# Supplementary material for: Reconciling fossils with phylogenies reveals the origin and macroevolutionary processes explaining the global cycad biodiversity
Source: New Phytol. 2023 Jun 11;240(4):1616–35. doi: 10.1111/nph.19010 (PMC10953041; doi:10.1111/nph.19010)
Supplement: Supplementary file 1 — Fig. S1 Maximum‐likelihood phylogeny of Cycadales. Fig. S2 Node support for phylogenetic analyses of Cycadales. Fig. S3 Bayesian chronogram from the total‐evidence dating analysis including extant and extinct taxa. Fig. S4 Bayesian chronogram from the total‐evidence dating analysis excluding the fossil taxa. Fig. S5 Phylogenetic placements of fossil cycads illustrated using RoguePlots. Fig. S6 Estimate of the historical biogeography for Cycadales with extant and extinct species. Fig. S7 Estimate of the historical biogeography for Cycadales with extant species only. Fig. S8 Estimate of the historical biogeography for Cycadales using BioGeoBEARS (DEC model) with extant and extinct species by coding fossil geographic ranges with missing data instead of true absences (option useAmbiguities = TRUE). Fig. S9 Estimate of the historical biogeography for Cycadales using BioGeoBEARS (DEC model) with extant and extinct species by coding fossil geographic ranges as true absences (option useAmbiguities = FALSE). Fig. S10 Estimate of the historical biogeography for Cycadales using BioGeoBEARS (DEC model) with extant and extinct species by taking into account the uncertainties in fossil placements and divergence times, and coding fossil geographic ranges with missing data (option useAmbiguities = TRUE). Fig. S11 Number of local extinctions (extirpations) per time bin (Cenozoic vs Mesozoic and Paleozoic) compared between analyses excluding fossils and analyses including fossils. Methods S1 Examination of fossil specimens. Notes S1 Morphological characters used in the total‐evidence dating analyses. Table S1 Specimens re‐examined during the coding of the matrix in this study. Table S2 Fossil species used in this study, and references for ages and morphology. Please note: Wiley is not responsible for the content or functionality of any Supporting Information supplied by the authors. Any queries (other than missing material) should be directed to the New Phytologist Central Office. [file NPH-240-1616-s001.pdf]

New Phytologist Supporting Information

Article title: Reconciling fossils with phylogenies reveals the origin and macroevolutionary processes explaining the global cycad biodiversity

Authors: Mario Coiro, Rémi Allio, Nathan Mazet, Leyla J. Seyfullah, Fabien L. Condamine

Article acceptance date: 2nd of May 2023

Fig. S1 Maximum-likelihood phylogeny of Cycadales. This phylogeny was inferred with IQ-TREE using a traditional partitioning strategy and ultrafast bootstraps to estimate node supports (values ≥ 95 are considered as strong support). The outgroup (*Ginkgo biloba*) is removed.

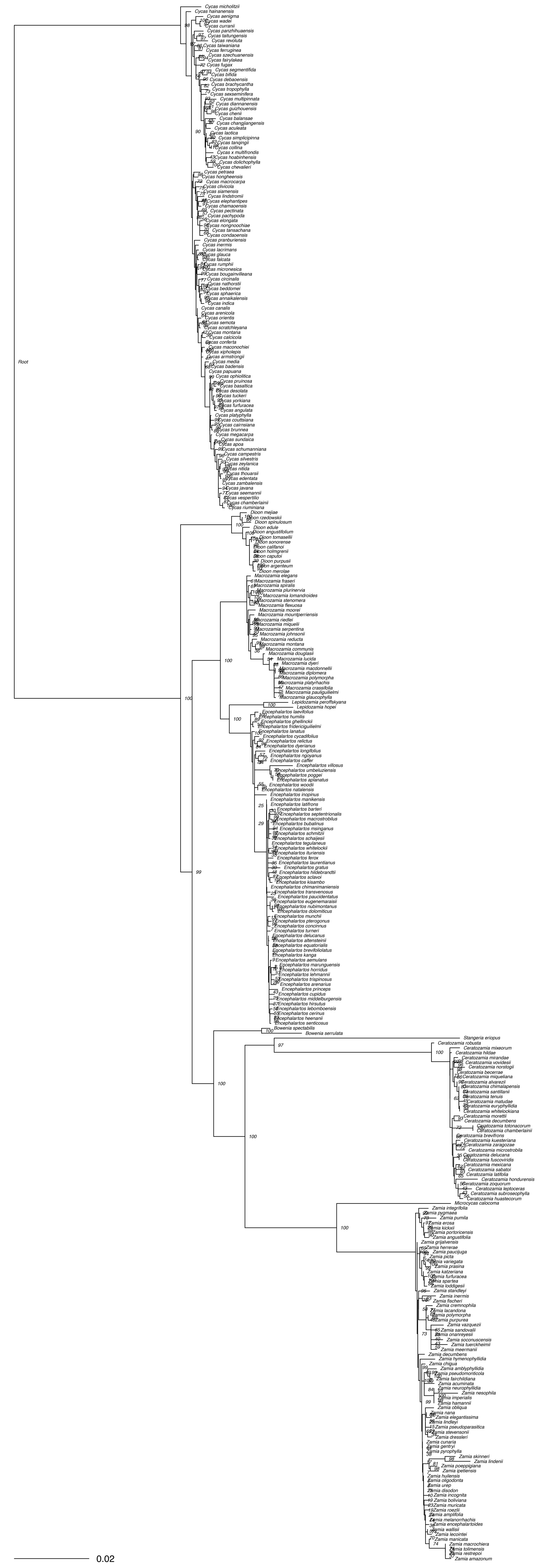
Root
0.02

Fig. S2 Node support for phylogenetic analyses of Cycadales. Histograms show that the phylogeny of Cycadales is generally robust, but many nodes within the extant genera remain unresolved. Percentages of strongly supported nodes and nodes with maximal support as well as mean and median node supports are reported for the IQ-TREE analysis with corresponding thresholds considered as strong supports.

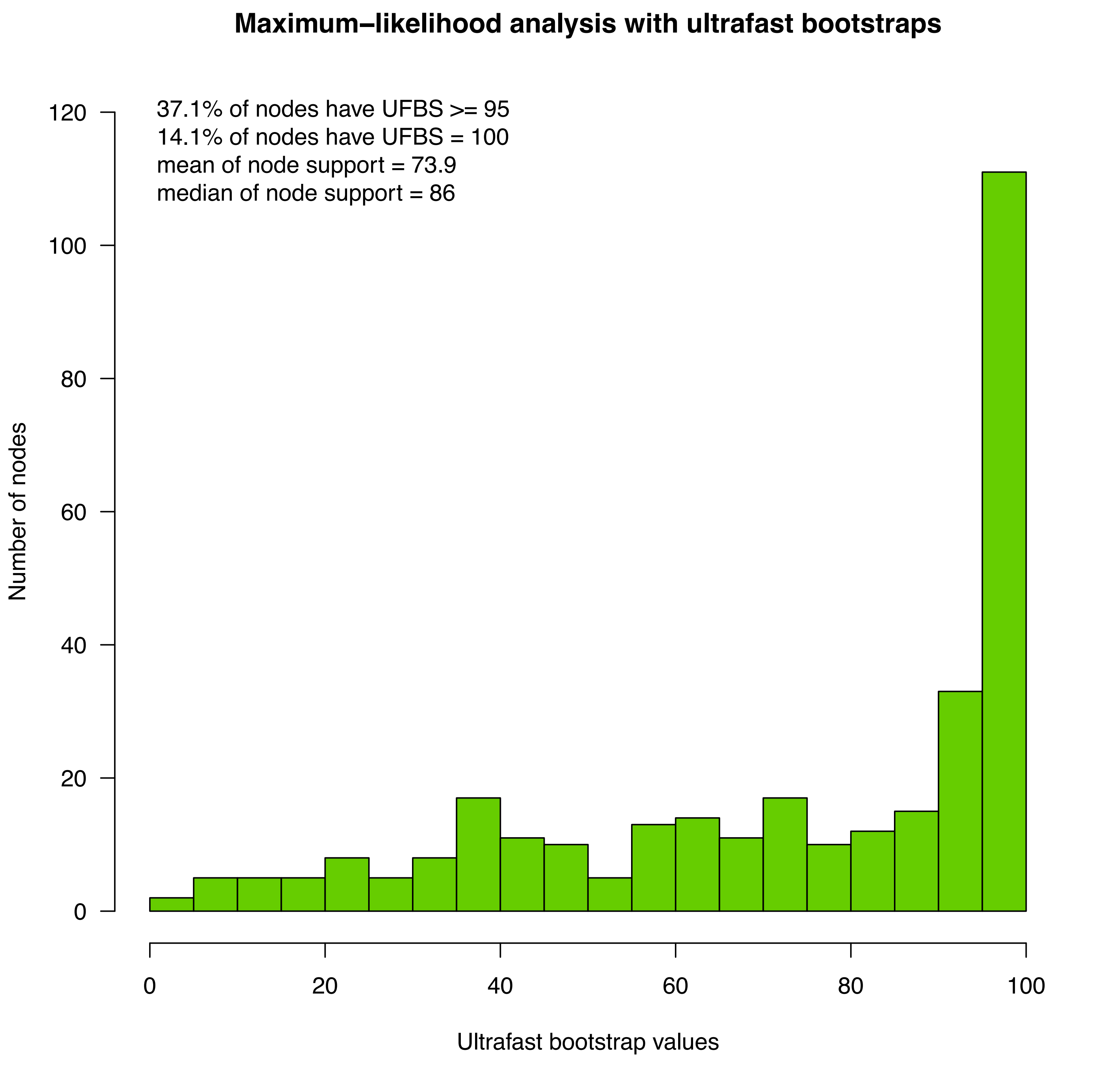

Maximum−likelihood analysis with ultrafast bootstraps
37.1% of nodes have UFBS >= 95
14.1% of nodes have UFBS = 100
mean of node support = 73.9
median of node support = 86
Number of nodes
0
20
40
60
80
100
120
0
20
40
60
80
100
Ultrafast bootstrap values

Fig. S3 Bayesian chronogram from the total-evidence dating analysis including extant and extinct taxa. The time-calibrated tree was inferred with MrBayes with the same partitioning strategy used for phylogenetic reconstructions and an uncorrelated lognormal clock model. Time is millions of years.

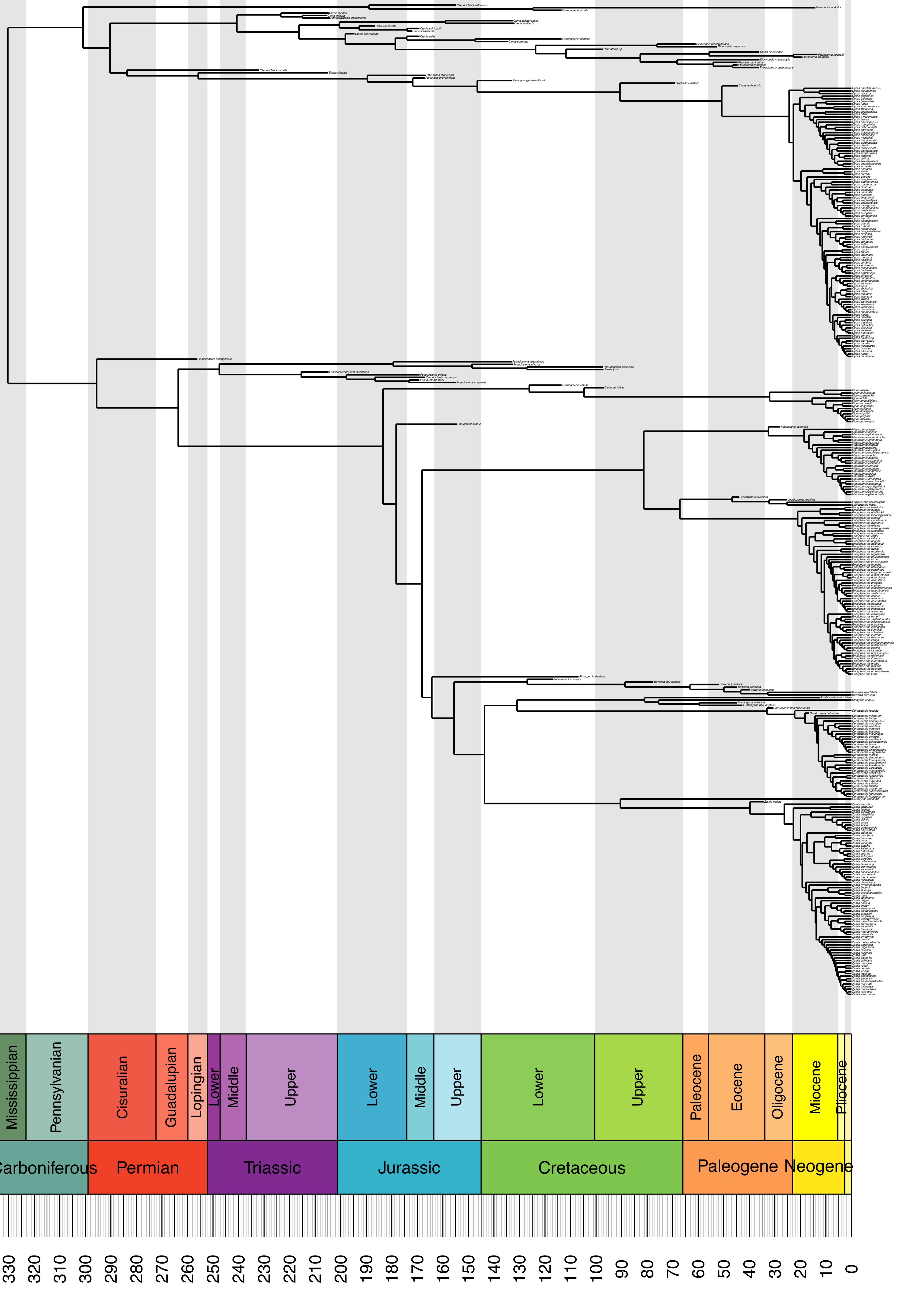
Mississippian
Pennsylvanian
Cisuralian
Guadalupian
Lopingian
Lower
Middle
Upper
Lower
Middle
Upper
Lower
Upper
Paleocene
Eocene
Oligocene
Miocene
Pliocene
Carboniferous
Permian
Triassic
Jurassic
Cretaceous
Paleogene
Neogene
330
320
310
300
290
280
270
260
250
240
230
220
210
200
190
180
170
160
150
140
130
120
110
100
90
80
70
60
50
40
30
20
10
0

Fig. S4 Bayesian chronogram from the total-evidence dating analysis excluding the fossil taxa. The time-calibrated tree was inferred with MrBayes with the same partitioning strategy used for phylogenetic reconstructions and an uncorrelated lognormal clock model. Time is millions of years.

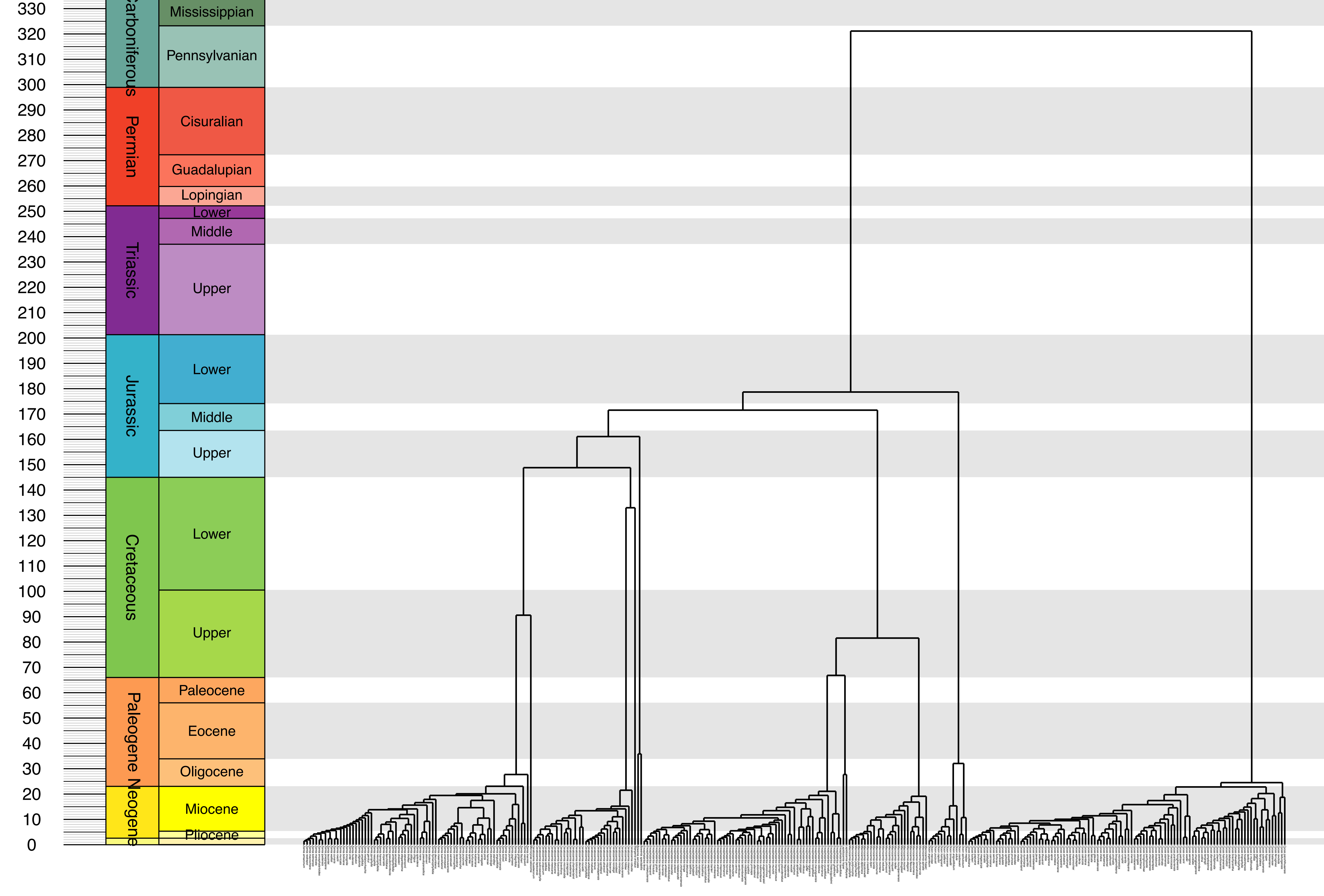
330
320
310
300
290
280
270
260
250
240
230
220
210
200
190
180
170
160
150
140
130
120
110
100
90
80
70
60
50
40
30
20
10
0
Carboniferous
Permian
Triassic
Jurassic
Cretaceous
Paleogene
Neogene
Mississippian
Pennsylvanian
Cisuralian
Guadalupian
Lopingian
Lower
Middle
Upper
Lower
Middle
Upper
Lower
Upper
Paleocene
Eocene
Oligocene
Miocene
Pliocene

Fig. S5 Phylogenetic placements of fossil cycads illustrated using RoguePlots. Here are shown members of the genus *Dioonopsis* (left) and *Eostangeria* (right). RoguePlots are trimmed to include only clades including branches with PP>0.08.

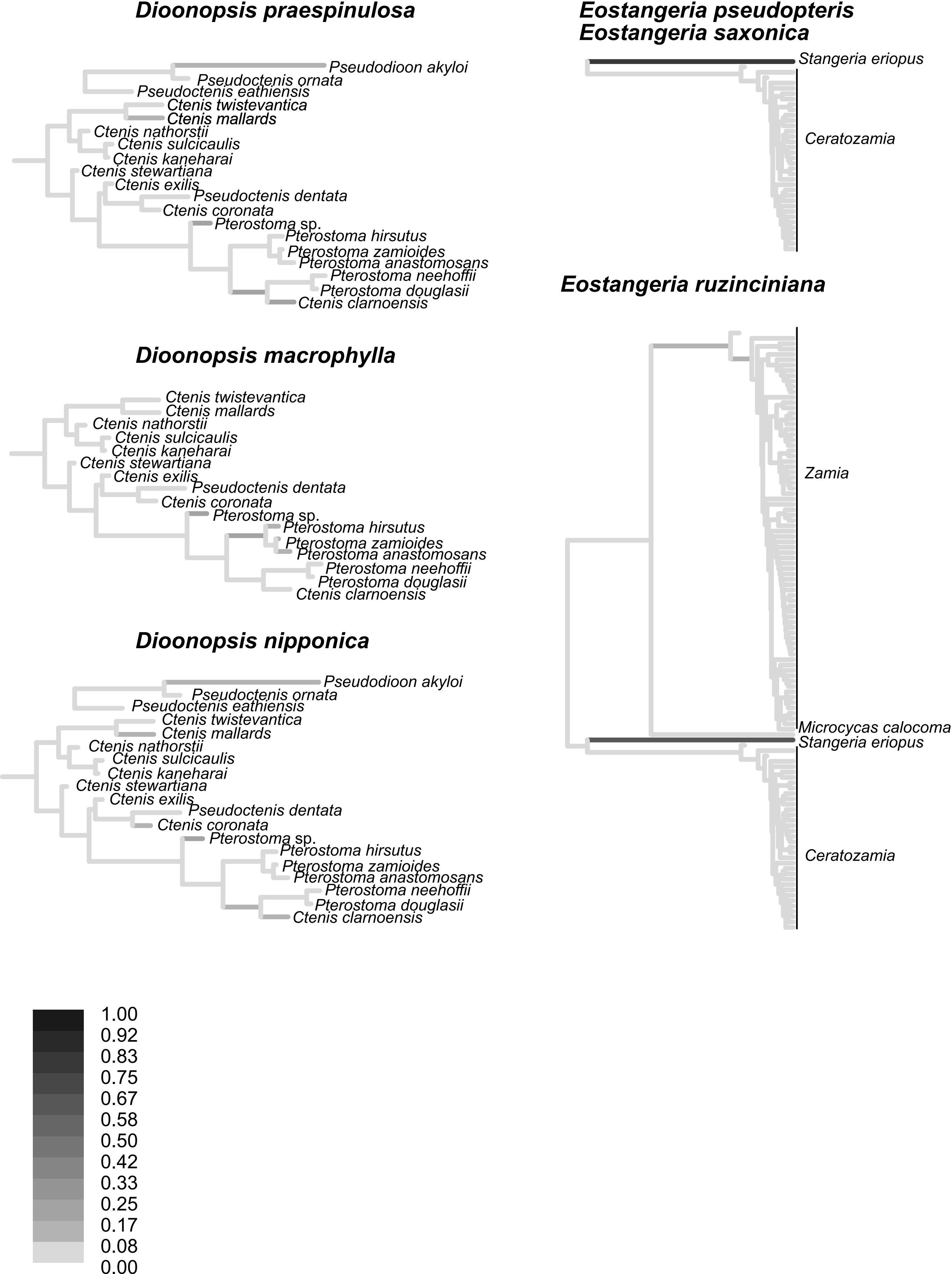

Dioonopsis praespinulosa
Pseudodioon akyloi
Pseudoctenis ornata
Pseudoctenis eathiensis
Ctenis twistevantica
Ctenis mallards
Ctenis nathorstii
Ctenis sulcicaulis
Ctenis kaneharai
Ctenis stewartiana
Ctenis exilis
Pseudoctenis dentata
Ctenis coronata
Pterostoma sp.
Pterostoma hirsutus
Pterostoma zamioides
Pterostoma anastomosans
Pterostoma neehoffii
Pterostoma douglasii
Ctenis clarnoensis
Dioonopsis macrophylla
Dioonopsis nipponica
Eostangeria pseudopteris
Eostangeria saxonica
Stangeria eriopus
Ceratozamia
Eostangeria ruzinciniana
Zamia
Microcycas calocoma
1.00
0.92
0.83
0.75
0.67
0.58
0.50
0.42
0.33
0.25
0.17
0.08
0.00

Fig. S6 Estimate of the historical biogeography for Cycadales with extant and extinct species. The Dispersal-Extinction-Cladogenesis (DEC) model was applied with time-stratified paleogeographic constraints. J=Jurassic, K=Cretaceous, Pal.=Paleocene, Oli.=Oligocene, Mioc.=Miocene. Pliocene and Pleistocene are the last two epochs but are not labelled. Given the tree size, one can zoom in across the tree to read details, particularly within extant genera. Red crosses indicate geographic extinction, and black circles around nodes denote vicariance events.

Historical biogeography of Cycadales (fossils included)

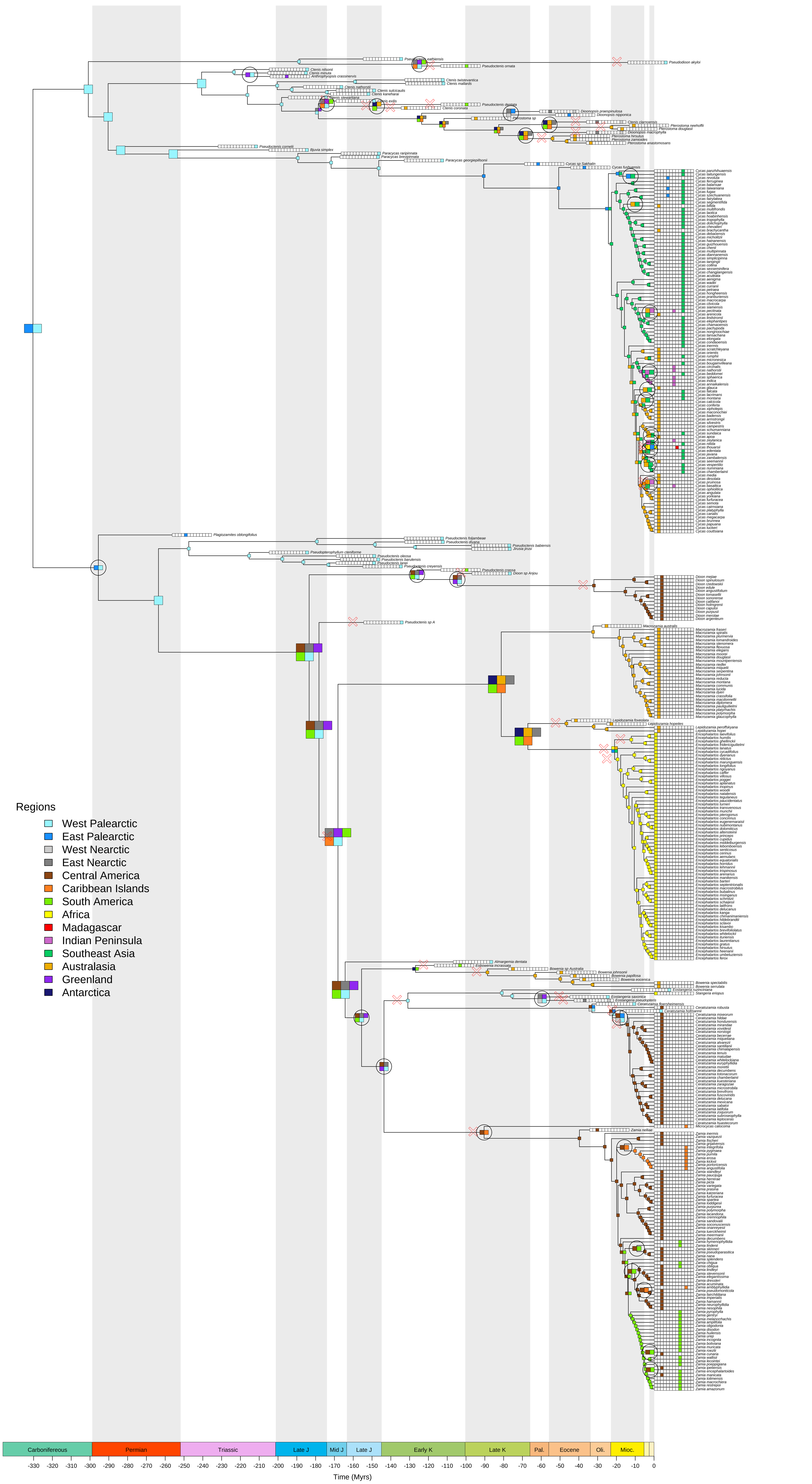

Fig. S7 Estimate of the historical biogeography for Cycadales with extant species only. The Dispersal-Extinction-Cladogenesis (DEC) model was applied with time-stratified paleogeographic constraints. J=Jurassic, K=Cretaceous, Pal.=Paleocene, Oli.=Oligocene, Mioc.=Miocene. Pliocene and Pleistocene are the last two epochs but are not labelled. Given the tree size, one can zoom in across the tree to read details, particularly within extant genera. Red crosses indicate geographic extinction, and black circles around nodes denote vicariance events.

Historical biogeography of Cycadales (fossils excluded)

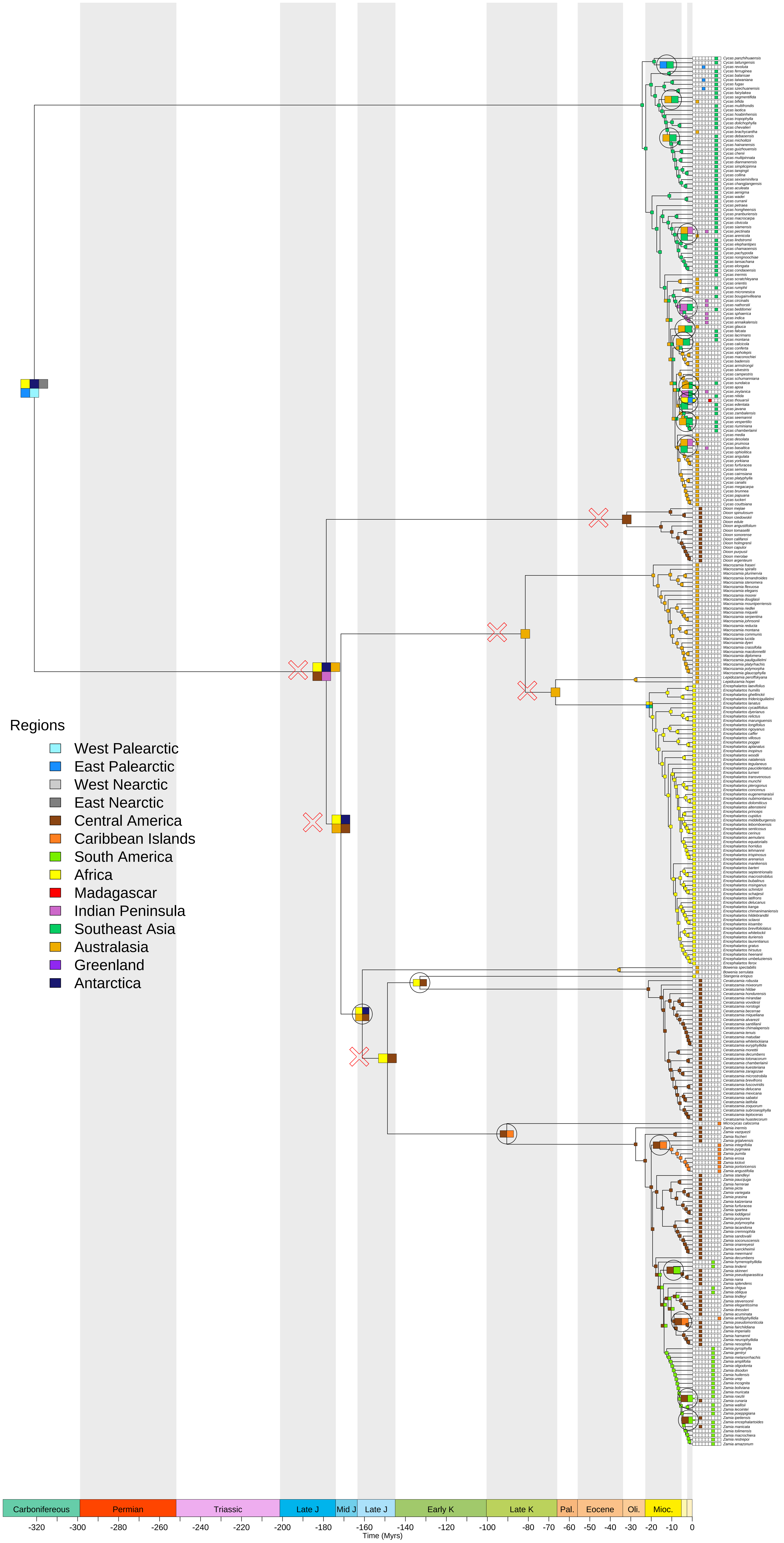

Fig. S8 Estimate of the historical biogeography for Cycadales using BioGeoBEARS (DEC model) with extant and extinct species by coding fossil geographic ranges with missing data instead of true absences (option *useAmbiguities=TRUE*).

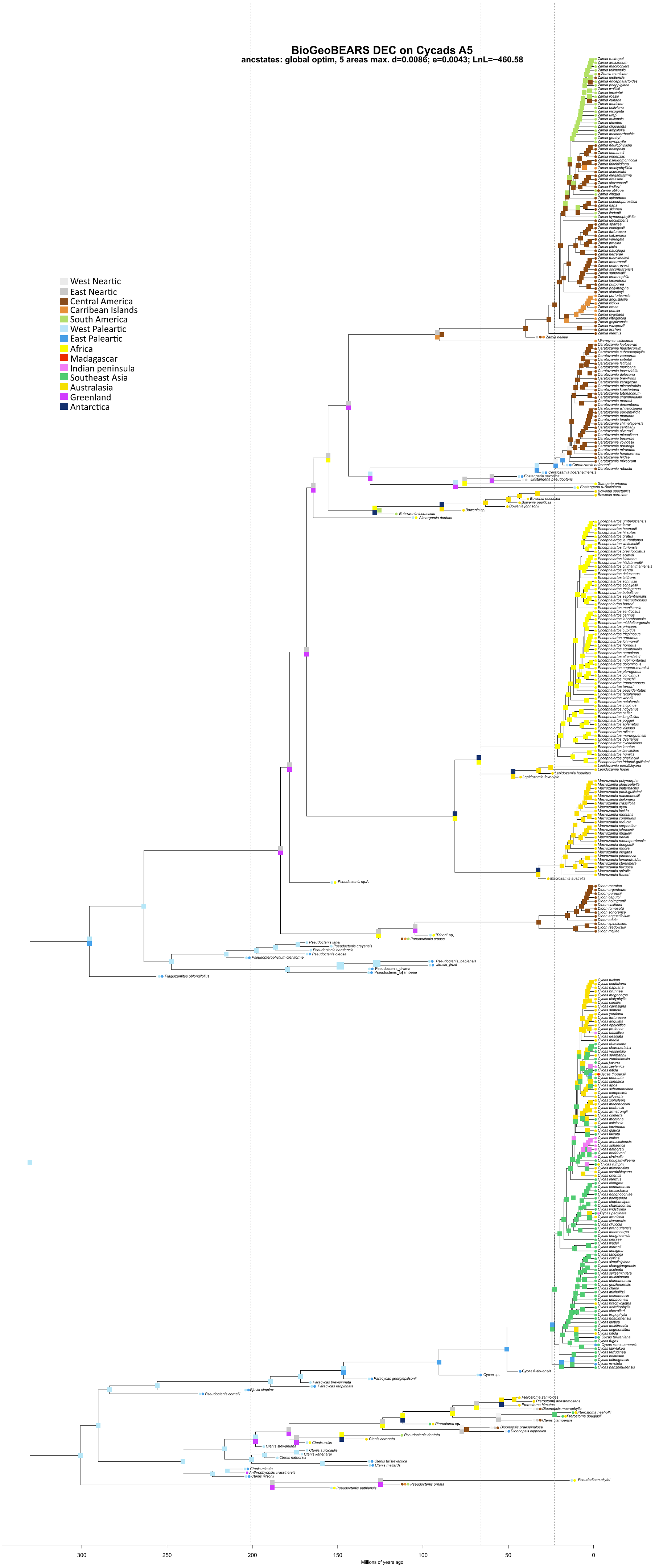

BioGeoBEARS DEC on Cycads A5
ancstates: global optim, 5 areas max. d=0.0086; e=0.0043; LnL=−460.58
West Neartic
East Neartic
Central America
Carribean Islands
South America
West Paleartic
East Paleartic
Africa
Madagascar
Indian peninsula
Southeast Asia
Australasia
Greenland
Antarctica
300
250
200
150
100
50
0
Millions of years ago

Fig. S9 Estimate of the historical biogeography for Cycadales using BioGeoBEARS (DEC model) with extant and extinct species by coding fossil geographic ranges as true absences (option *useAmbiguities=FALSE*).

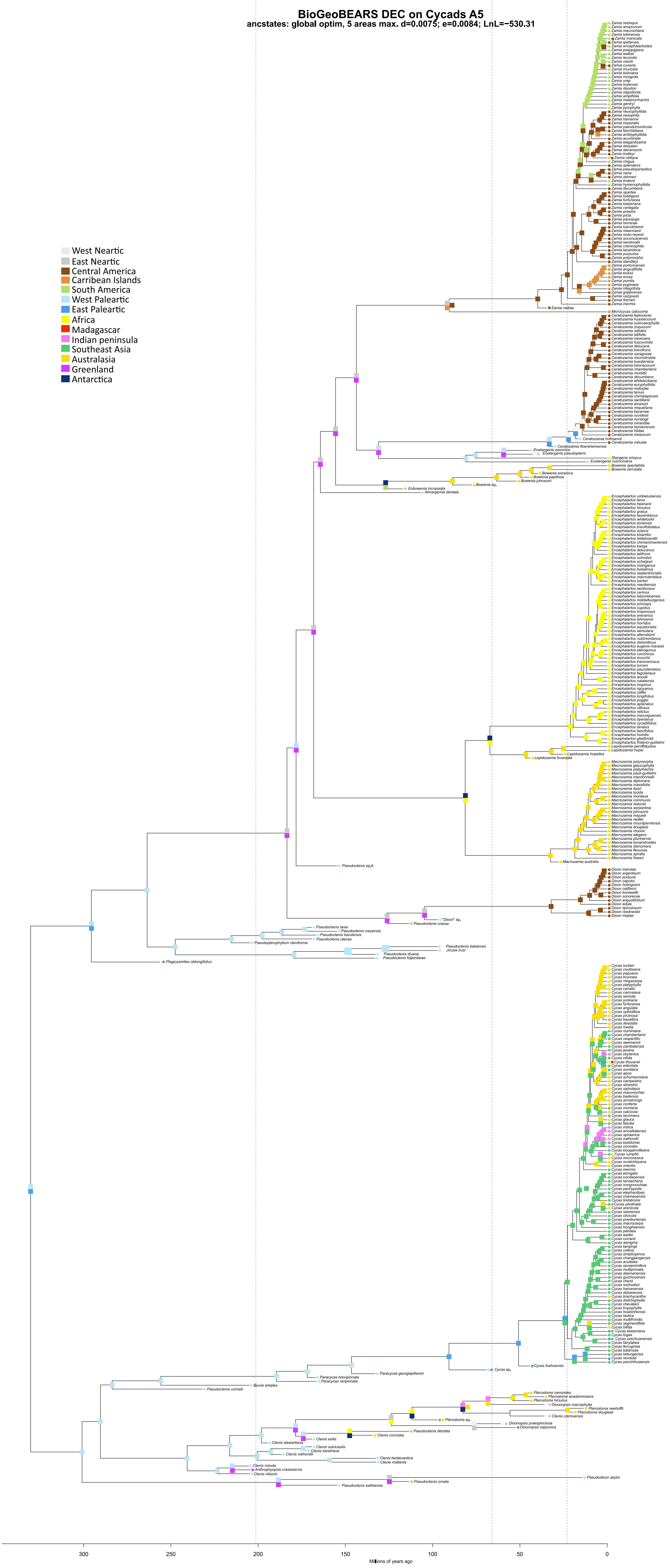
BioGeoBEARS DEC on Cycads A5
ancstates: global optim, 5 areas max. d=0.0075; e=0.0084; LnL=−530.31
West Neartic
East Neartic
Central America
Carribean Islands
South America
West Palearctic
East Palearctic
Africa
Madagascar
Indian peninsula
Southeast Asia
Australasia
Greenland
Antarctica
300
250
200
150
100
50
0
Millions of years ago

Fig. S10 Estimate of the historical biogeography for Cycadales using BioGeoBEARS (DEC model) with extant and extinct species by taking into account the uncertainties in fossil placements and divergence times, and coding fossil geographic ranges with missing data (option *useAmbiguities=TRUE*).

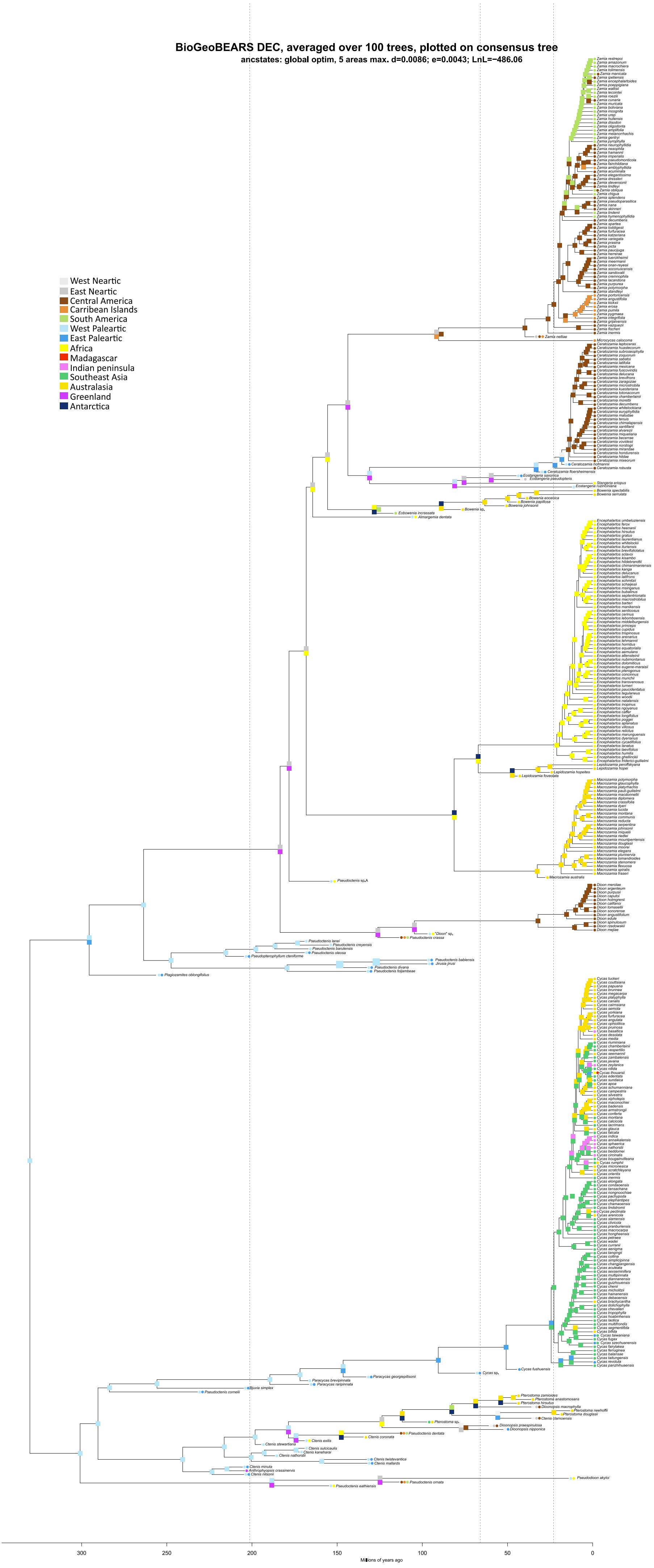
BioGeoBEARS DEC, averaged over 100 trees, plotted on consensus tree
ancstates: global optim, 5 areas max. d=0.0086; e=0.0043; LnL=−486.06
West Neartic
East Neartic
Central America
Carribean Islands
South America
West Paleartic
East Paleartic
Africa
Madagascar
Indian peninsula
Southeast Asia
Australasia
Greenland
Antarctica
300
250
200
150
100
50
0
Millions of years ago

Fig. S11 Number of local extinctions (extirpations) per time bin (Cenozoic vs. Mesozoic and Paleozoic) compared between analyses excluding fossils (A and B) and analyses including fossils (C and D). Area names: WP=West Palearctic, EP=East Palearctic, WN=West Nearctic, EN=East Nearctic, CA=Central America, WI=Caribbean Islands, SA=South America, AF=Africa, IN=India, WA=Southeast Asia, AU=Australasia, GR=Greenland, and AN=Antarctica.

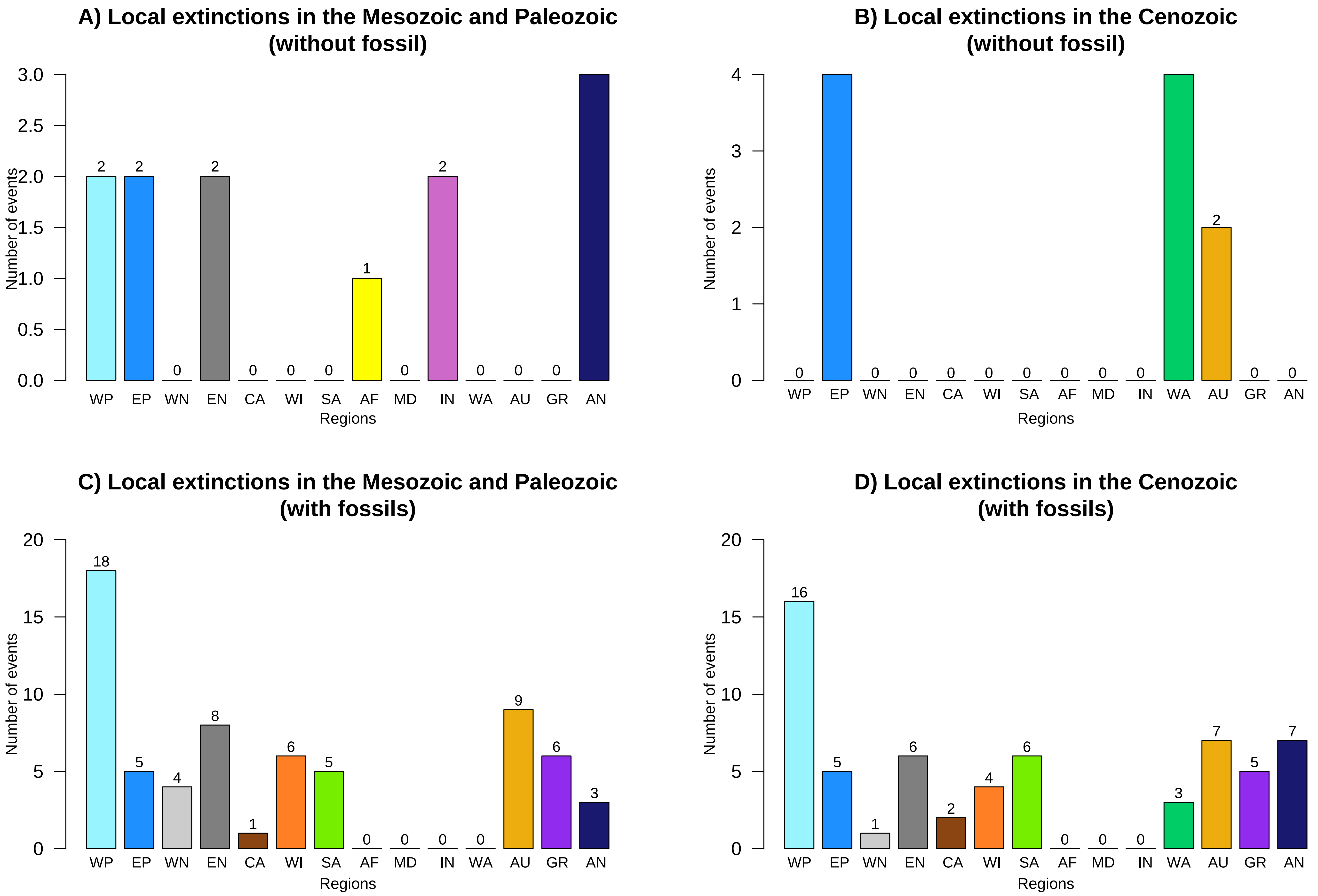

A) Local extinctions in the Mesozoic and Paleozoic (without fossil)
B) Local extinctions in the Cenozoic (without fossil)
C) Local extinctions in the Mesozoic and Paleozoic (with fossils)
D) Local extinctions in the Cenozoic (with fossils)
Number of events
Regions
WP EP WN EN CA WI SA AF MD IN WA AU GR AN

Supplementary Methods S1 Examination of fossil specimens.

To better understand the morphology of fossil cycad leaves and build our morphological matrix, we observed numerous specimens held at the Natural History Museum London and the Stockholm National Museum of Natural History. Macrofossil specimens were observed using a stereomicroscope, while cuticles were observed using either a Nikon Eclipse LV100ND microscope or an Olympus BX-51 light microscope, which was modified for epifluorescence microscopy, and were photographed with an Olympus DP-71 digital camera. Specimens are listed in Table S1. We selected only characters that were possible to score for both extant and extinct cycads (see Note S1). The widespread Mesozoic genus *Nilssonia* Brongniart was not included because there are doubts concerning its cycadalean affinity to Cycadales and its highly unstable position in preliminary analyses.

Table S1. Specimens re-examined during the coding of the matrix in this study.

Species	Specimen	Type	Museum
Almargemia dentata	S085614	Cuticle	NRM-SE
Almargemia dentata	S085615	Cuticle	NRM-SE
Almargemia dentata	S085616	Cuticle	NRM-SE
Almargemia dentata	S085617	Cuticle	NRM-SE
Almargemia dentata	S085618	Cuticle	NRM-SE
Almargemia dentata	S085619	Cuticle	NRM-SE
Almargemia dentata	S085620	Cuticle	NRM-SE
Almargemia dentata	S085621	Cuticle	NRM-SE
Bjuvia simplex	S055058	Macrofossil	NRM-SE
Bjuvia simplex	S055059	Macrofossil	NRM-SE
Bjuvia simplex	S055059-02	Cuticle	NRM-SE
Bjuvia simplex	S055059-03	Cuticle	NRM-SE
Bjuvia simplex	S055059-04	Cuticle	NRM-SE
Bjuvia simplex	S055059-05	Cuticle	NRM-SE
Bjuvia simplex	S055059-08	Cuticle	NRM-SE
Bjuvia simplex	S055468	Macrofossil	NRM-SE
Bjuvia simplex	S055469	Macrofossil	NRM-SE
Ctenis exilis	v45612	Cuticle	NHM-GB
Ctenis exilis	v45613	Cuticle	NHM-GB
Ctenis exilis	v45614	Cuticle	NHM-GB
Ctenis exilis	v45615	Cuticle	NHM-GB
Ctenis exilis	v45616	Cuticle	NHM-GB
Ctenis exilis	v45617	Cuticle	NHM-GB
Ctenis exilis	v45618	Cuticle	NHM-GB
Ctenis exilis	v45619	Cuticle	NHM-GB
Ctenis exilis	v45620	Cuticle	NHM-GB
Ctenis exilis	v45621	Cuticle	NHM-GB
Ctenis exilis	v45622	Cuticle	NHM-GB
Ctenis kaneharai	V28288	Macrofossil	NHM-GB
Ctenis kaneharai	V28289	Macrofossil	NHM-GB
Ctenis kaneharai	V28290	Macrofossil	NHM-GB
Ctenis kaneharai	V28291	Macrofossil	NHM-GB
Ctenis kaneharai	V28292	Macrofossil	NHM-GB
Ctenis kaneharai	V28293	Macrofossil	NHM-GB
Ctenis kaneharai	v28394	Cuticle	NHM-GB
Ctenis kaneharai	v44865	Macrofossil	NHM-GB
Ctenis kaneharai	v45623	Cuticle	NHM-GB
Ctenis kaneharai	v45624	Cuticle	NHM-GB
Ctenis kaneharai	v45625	Cuticle	NHM-GB
Ctenis kaneharai	v45626	Cuticle	NHM-GB
Ctenis kaneharai	v45627	Cuticle	NHM-GB
Ctenis kaneharai	v45628	Cuticle	NHM-GB
Ctenis kaneharai	v45629	Cuticle	NHM-GB
Ctenis kaneharai	v45630	Cuticle	NHM-GB
Ctenis kaneharai	v45631	Cuticle	NHM-GB
Ctenis kaneharai	v45636	Cuticle	NHM-GB

Ctenis kaneharai	v45637	Cuticle	NHM-GB
Ctenis latepinnata	S055471	Macrofossil	NRM-SE
Ctenis latepinnata	S055471-01	Cuticle	NRM-SE
Ctenis latepinnata	S055471-02	Cuticle	NRM-SE
Ctenis laxa	S055474	Macrofossil	NRM-SE
Ctenis laxa	S055475	Macrofossil	NRM-SE
Ctenis laxa	S057120-01	Cuticle	NRM-SE
Ctenis laxa	S057120-02	Cuticle	NRM-SE
Ctenis laxa	S057120-03	Cuticle	NRM-SE
Ctenis minuta	S054937	Macrofossil	NRM-SE
Ctenis minuta	S054937-01	Cuticle	NRM-SE
Ctenis minuta	S057108	Cuticle	NRM-SE
Ctenis minuta	S057112	Cuticle	NRM-SE
Ctenis minuta	S057113	Cuticle	NRM-SE
Ctenis nathorstii	S085627	Cuticle	NRM-SE
Ctenis nathorstii	S085629	Cuticle	NRM-SE
Ctenis nathorstii	S170556	Macrofossil	NRM-SE
Ctenis nathorstii	S170558	Macrofossil	NRM-SE
Ctenis nathorstii	S170559	Macrofossil	NRM-SE
Ctenis nilssonii	S054934	Cuticle	NRM-SE
Ctenis nilssonii	S054934-04	Cuticle	NRM-SE
Ctenis nilssonii	S055444	Macrofossil	NRM-SE
Ctenis nilssonii	S055445	Macrofossil	NRM-SE
Ctenis nilssonii	S055446	Macrofossil	NRM-SE
Ctenis nilssonii	S055447	Macrofossil	NRM-SE
Ctenis nilssonii	S057126	Cuticle	NRM-SE
Ctenis stewartiana	S067096	Macrofossil	NRM-SE
Ctenis stewartiana	S067177	Macrofossil	NRM-SE
Ctenis stewartiana	S067179	Cuticle	NRM-SE
Ctenis stewartiana	S067513	Macrofossil	NRM-SE
Ctenis stewartiana	v45638	Cuticle	NHM-GB
Ctenis sulcicaulis	8089	Macrofossil	NHM-GB
Ctenis sulcicaulis	38763	Macrofossil	NHM-GB
Ctenis sulcicaulis	39205	Macrofossil	NHM-GB
Ctenis sulcicaulis	S150221	Cuticle	NRM-SE
Ctenis sulcicaulis	v24671	Macrofossil	NHM-GB
Ctenis sulcicaulis	v25865	Macrofossil	NHM-GB
Ctenis sulcicaulis	v25879	Macrofossil	NHM-GB
Ctenis sulcicaulis	v38899	Cuticle	NHM-GB
Ctenis sulcicaulis	v44856	Macrofossil	NHM-GB
Ctenis sulcicaulis	v44857	Macrofossil	NHM-GB
Ctenis sulcicaulis	v44858	Macrofossil	NHM-GB
Ctenis sulcicaulis	v45639	Cuticle	NHM-GB
Ctenis sulcicaulis	v45640	Cuticle	NHM-GB
Ctenis sulcicaulis	v45641	Cuticle	NHM-GB
Ctenis sulcicaulis	v45642	Cuticle	NHM-GB
Ctenis sulcicaulis	v45643	Cuticle	NHM-GB
Ctenis sulcicaulis	v45644	Cuticle	NHM-GB
Ctenis sulcicaulis	v45645	Cuticle	NHM-GB

Ctenis sulcicaulis	v45646	Cuticle	NHM-GB
Ctenis sulcicaulis	v58901	Macrofossil	NHM-GB
Ctenis sulcicaulis	v60150	Macrofossil	NHM-GB
Eobowenia incrassata	v52264	Macrofossil	NHM-GB
Eobowenia incrassata	v52265	Cuticle	NHM-GB
Pseudoctenis lanei	v28302	Cuticle	NHM-GB
Pseudoctenis lanei	v42391	Cuticle	NHM-GB
Pseudoctenis lanei	v60157a	Cuticle	NHM-GB
Pseudoctenis lanei	v60157b	Cuticle	NHM-GB
Pseudoctenis oleosa	v27713b	Cuticle	NHM-GB
Pseudoctenis oleosa	v27714a	Cuticle	NHM-GB
Pseudoctenis oleosa	v60156a	Cuticle	NHM-GB
Pseudoctenis oleosa	v60156b	Cuticle	NHM-GB
Pseudopterophyllum cteniforme	S054980	Macrofossil	NRM-SE
Pseudopterophyllum cteniforme	S054980-04	Cuticle	NRM-SE
Pseudopterophyllum cteniforme	S054980-06	Cuticle	NRM-SE
Pseudopterophyllum cteniforme	S054980-09	Cuticle	NRM-SE

Table S2. Fossil species used in this study, and references for ages and morphology.

Taxon name	Locality	Period	Age range (Ma)	Reference
Almargemia dentata Florin	Portugal	Cretaceous	100.5-113.0	Florin (1933); Coiro & Pott (2017)
Anthrophyopsis crassinervis Harris	Greenland	Triassic	201.3-208.5	Harris (1932),personal observations
Bjuvia simplex Florin	Sweden	Triassic	201.3-208.5	Florin (1933), personal observations
Bowenia eocenica Hill	Australia	Paleogene	38.0-41.3	Hill (1980)
Bowenia johnsonii	Australia	Paleogene	47.8-56	Hill *et al.* (2019)
Bowenia papillosa Hill	Australia	Palaeogene	41.3-47.8	Hill (1980)
Bowenia sp.	Australia	Cretaceous	72.1-83.6	Carpenter *et al.* (2015)
Ceratozamia floersheimensis Kvaček	Central Europe	Paleogene	28.1-33.9	Kvaček (2002)
Ceratozamia hofmannii Kvaček	Central Europe	Neogene	16.5-16.7	Kvaček (2014)
Ctenis clarnoensis B.Erdei & Manchester	US	Paleogene	33.9-38.0	Erdei & Manchester (2015)
Ctenis coronata Douglas	Australia	Cretaceous	129.4-139.8	Douglas (1969)
Ctenis exilis Harris	England	Jurassic	168- 170.3	Harris (1964), personal observations
Ctenis kaneharai Yokoyama	England	Jurassic	170.3-174.1	Harris (1964), personal observations
Ctenis mallards Watson & Cusack	England	Cretaceous	125.0-139.8	Watson and Cusack (2005)
Ctenis minuta Florin	Sweden	Triassic	201.3-208.5	Florin (1933), personal observations
Ctenis nathorsti Moeller	Denmark	Jurassic	182.7-190.8	Florin (1933), personal observations
Ctenis nilsonii (Nath.) Harris	Sweden	Triassic	201.3-208.5	Florin (1933), personal observations
Ctenis stewartiana Harris	England	Jurassic	190.8-199.3	Florin (1933), Harris (1964), personal observations
Ctenis sulcicaulis (Phillips) Ward.	England	Jurassic	168- 170.3	Harris (1964), personal observations
Ctenis twistevanica	England	Cretaceous	125.0-139.8	Watson & Cusack (2005)
Cycas fushunensis Su, Quan & Lu	China	Paleogene	41.3-47.8	Su *et al.* (2014)
Cycas sp.	Russia	Cretaceous	66-72.1	Krassilov (1978)
Dioon sp.	France	Cretaceous	93.9–100.5	Berthelin & Pons (1999)
Dioonopsis macrophylla (Potbury)	US	Palaeogene	33.9-38.0	Erdei *et al.* (2012)
Dioonopsis nipponica Horiuchi & Kimura	Japan	Paleogene	23-66	Horiuchi & Kimura (1987)
Dioonopsis praespinulosa (Hollick) Erdei, Manchester & Kvaček	US	Paleogene	56-66	Erdei *et al.* (2012)
Eobowenia incrassata M.Coiro & C.Pott	Argentina	Cretaceous	113-114	Coiro and Pott (2017)
Eostangeria pseudopteris Kvaček & Manchester	US	Paleogene	38-47.8	Kvaček & Manchester, (1999)
Eostangeria saxonica Barthel	Germany	Paleogene	41.3-47.8	Barthel, (1976)
Eostangeria ruzinciniana (Palamarev, Petkova & Uzunova) Palamarev & Uzunova	Bulgaria	Neogene	11.6-12.7	Uzunova *et al.* (2001)

Lepidozamia foveolata Hill	Australia	Paleogene	41.3-47.8	Hill (1980)
Lepidozamia hopeites (Cookson) L. Johnson	Australia	Paleogene/Neogene	15.9-33.9	Cookson (1953)
Jirusia jirusii (Bayer) Domin.	Czech Republic	Cretaceous	93.9–100.5	Kvaček (1995)
Macrozamia australis Carpenter	Australia	Paleogene	23.03-33.9	Carpenter (1991)
Paracycas brevipinnata Delle	Georgia	Jurassic	163.5-170.3	Doludenko & Svanidze (1969)
Paracycas raripinnata Dolud.	Georgia	Jurassic	163.5-170.3	Doludenko & Svanidze (1969)
Pseudoctenis babinensis J.Kvaček	Czech Republic	Cretaceous	93.9–100.5	Kvaček (2008)
Pseudoctenis barulensis Dolud.	Georgia	Jurassic	163.5-170.3	Doludenko & Svanidze (1969)
Pseudoctenis crassa S.Archang	Argentina	Cretaceous	113-114	Archangelsky & Baldoni (1972)
Pseudoctenis creysensis Barale	France	Jurassic	152.1-157.3	Barale (1981)
Pseudoctenis dentata Archangelsky & Baldoni	Argentina	Cretaceous	113-114	Archangelsky & Baldoni (1972), Passalia (2013)
Pseudoctenis eathiensis (Richards) Seward	Scotland	Jurassic	152.1-157.3	Van Konijnenburg-Van Cittert & Van Der Burgh (1989)
Pseudoctenis lanei Thomas	England	Jurassic	168- 170.3	Harris (1964), personal observations
Pseudoctenis latus Dolud.	Georgia	Jurassic	163.5-170.3	Doludenko & Svanidze (1969)
Pseudoctenis oleosa Harris	England	Jurassic	168- 170.3	Harris (1964), personal observations
Pseudoctenis ornata A.Archang., R.Andreis, S.Archang. &. A.Artabe	Argentina	Cretaceous	113-114	Archangelsky *et al.* (1995)
Pseudoctenis sp. A	France	Jurassic	152.1-157.3	Barale (1981)
Pseudodioon akyoli Erdei, Akgun & Barone Lumaga	Turkey	Neogene	11.6-15.97	Erdei *et al.* (2009)
Pseudopterophyllum cteniforme (Nathorst) Florin	Sweden	Triassic	201.3-208.5	Florin (1933), personal observations
Pterostoma anastomosans Hill	Australia	Paleogene	33.9-38.0	Hill (1980)
Pterostoma douglasii Hill & Pole	New Zealand	Neogene	15.97-23.03	Hill & Pole (1994)
Pterostoma hirsutus Hill & Pole	Australia	Paleogene	41.3-47.8	Hill & Pole (1994)
Pterostoma zamioides Hill	Australia	Paleogene	41.3-47.8	Hill (1980)
Pterostoma sp	Australia	Cretaceous	93.9-100.5	Pole & Douglas (1999)
Pterostoma nehoffii	New Zealand	Paleogene	23.03-33.9	Conran *et al.* (2020)
Plagiozamites oblongifolius Halle	China	Permian	254.14-259.1	Feng *et al.* (2017)
Pseudoctenis cornelii Pott, Kerp & Krings	Austria	Triassic	227-237	Pott *et al.* (2007)
Zamia nelliae Erdei & Calonje	Panama	Paleogene	33-35	Erdei *et al.* (2018)

Note S1. Morphological characters used in the total-evidence dating analyses.

Character 1: Lamina attachment: (0) adaxial, (1) medial. From character 26 of Martinez *et al.* (2012).

Character 2: Leaf architecture (0) Once pinnate (1) Bipinnate (2) Taeniopteroid. Recodified from character 24 of Martinez *et al.* (2012).

Character 3: Leaflets (0) regular (1) irregular. This character was erected to distinguish the irregular, dissected leaflets present in *Nilssonia* from the more regular leaflets of other cycads.

Character 4: Leaflet insertion (0) decurrent (1) articulate. From character 27 of Martinez *et al.* (2012).

Character 5: Leaflet margin (0) entire (1) dentate (2) serrate. This character was erected to distinguish the different leaflet margins, which are commonly used to distinguish between genera (i.e. *Dioonopsis, Encephalartites*). We distinguish the non-vascularized teeth present in *Dioon* and *Encephalartos* from the vascularized teeth present in *Bowenia* and *Zamia*.

Character 6: Leaflet venation (0) Parallel veins (1) Single vein. Recodified from character 30 of Martinez *et al.* (2012).

Character 7:H-anatomoses (0) Absent (1) Present. This character was erected to distinguish the situation present in *Ctenis* and other fossil leaves, where the anastomoses between veins do not correspond to reduction in vein numbers.

Character 8: terminal anastomoses (0) absent (1) present. Recodified from character 33 of Martinez *et al.* (2012).

Character 9: Midvein in the leaflets (0) Present (1) Absent. This character distinguishes the multiveined leaves of *Stangeria* or *Zamia (Chigua) restrepoi*, which present a midrib, from all other multiveined leaves.

Character 10: Leaflet midvein type: (0) *Cycas*-type (1) *Stangeria*-type. Modified from character 32 of Martinez *et al.* (2012).

Character 11: Stomata (0) flush (1) sunken. Character 50 of Martinez *et al.* (2012).

Character 12: Stomatal orientation (0) longitudinal (1) random. Modified from character 52 of Martinez *et al.* (2012).

Character 13: Stomatal disposition (0) In bands between veins (1) diffuse (2) in groups. This distinguishes between taxa with no clear stomatal bands, taxa with stomatal bands and

taxa with clumps of stomata that do not correspond to intervein bands (such as *Ctenis minuta*).

Character 14: Coronal rim (0) absent (1) present. This structure is similar to the accessory cell corona of Martinez *et al.* (2012), i.e. a cuticular rim that surrounds the stomatal opening. It is typical of many *Ctenis* taxa.

Character 15: Substomatal complex (0) non thickened (1) thickened. Character 89 from Coiro and Pott (2017)

Character 16: Subsidiary cells (0) thick cuticle (1) thin cuticle. This character distinguishes the Zamiaceae from other cycads.

Character 17: Stomatal pit (0) absent (1) present. This character indicates the presence of a chamber formed by the encircling cells (like in *Dioon* or *Cycas revoluta*).

Character 18: Lateral encircling cells (0) zero (1) one (2) two (3) three. Modified from character 53 of Martinez *et al.* (2012).

Character 19: Papillae on the stomatal pit (0) absent (1) present.

Character 20: Polar encircling cells (0) absent (1) present. This character indicates the presence of differentiated polar encircling cells.

Character 21: Polar encircling cells overarching (0) absent (1) present. This character indicates whether the polar encircling cells elongate to overarch the poles of the guard cells.

Character 22: Polar cuticular extension (0) absent (1) present. This character describes the presence of a cuticular intrusion between the poles of the guard cells and the polar pavement cells.

Character 23: Anticlinal pegs (0) absent (1) present. Character 48 of Martinez *et al.* (2012).

Character 24 Epidermal cells in stomatal bands (0) elongated (1) isodiametrical. Elongated cells are typical of most extant Zamiaceae.

Character 25 Thin-walled cells (0) absent (1) present. Modified from character 46 of Martinez *et al.* (2012).

Character 26 Thin-walled cell files (0) on the costal epidermis (1) on the intercostal epidermis (2) absent. This character separates the typical cells files of *Ceratozamia* and *Dioon.*

Character 27 Anticlinal walls (0) straight (1) wavy.

Character 28 Trichome bases (0) circular (1) angular. Angular trichome bases are typical of *Cycas* and other fossil genera.

Character 29 Cuticular striae (0) absent (1) present. Modified from character 47 of Martinez *et al.* (2012).

Character 30 Epidermal idioblasts (0) absent (1) present. Epidermal idioblasts are typical of some species of *Dioon* and *Macrozamia*.

Character 31 Stomatal distribution (0) hypostomatic (1) amphistomatic. Character 49 of Martinez *et al.* (2012).

Supplementary References

Archangelsky A, Andreis RR, Archangelsky S, Artabe A. **1995**. Cuticular characters adapted to volcanic stress in a new Cretaceous cycad leaf from Patagonia , Argentina . Considerations on the stratigraphy and depositional history of the Baqueró Formation. *Review of Palaeobotany and Palynology* **89**: 213–233.

Archangelsky S, Baldoni A. **1972**. Notas sobre la flora de la zona de Tico, provincia de Santa Cruz. X. Dos nuevas especies de *Pseudoctenis* (Cycadales). *Ameghiniana* **9**: 241–257.

Barale G. **1981**. *La paléoflore jurassique du Jura français : étude systématique, aspects stratigraphiques et paléoécologiques*. Villeurbanne : Département des sciences de la terre, Université Claude-Bernard Lyon.

Barthel M. **1976**. Eozane Floren des Geiseltales: Farne und Cycadeen. *Abhandlungen des Zentralen Geologischen Institutes. Palaontol Abhandl* **26**: 439–498.

Berthelin M, Pons D. **1999**. Signification des caracteres partages entre Bennettitales et Cycadales. Implications de la decouverte d'une Cycadale nouvelle du Cenomanien de l'Anjou (France). *Annales de Paleontologie* **85**: 227–239.

Carpenter R. **1991**. *Macrozamia* from the early Tertiary of Tasmania and a study of the cuticles of extant species. *Australian Systematic Botany* **4**: 433–444.

Carpenter RJ, Macphail MK, Jordan GJ, Hill RS. **2015**. Fossil evidence for open, Proteaceae-dominated heathlands and fire in the Late Cretaceous of Australia. *American Journal of Botany* **102**: 1–16.

Coiro M, Pott C. **2017**. *Eobowenia* gen. nov. from the Early Cretaceous of Patagonia: Indication for an early divergence of *Bowenia*? *BMC Evolutionary Biology* **17**: 97.

Cookson IC. **1953**. On *Macrozamia hopeites* -an early Tertiary cycad from Australia. *Phytomorphology* **3**: 306–312.

Doludenko MP, Svanidze CI. **1969**. Pozdnejurskaja flora Gruzii. *Transactions of the Geological Institute, Academy of Sciences USSR* **178**: 1–116.

Douglas JG. **1969**. *The Mesozoic floras of Victoria*. Melbourne: Department of Mines.

Erdei B, Akgün F, Barone Lumaga MR. **2009**. *Pseudodioon akyoli* gen. et sp. nov., an extinct member of Cycadales from the Turkish Miocene. *Plant Systematics and Evolution* **285**: 33–49.

Erdei B, Calonje M, Hendy A, Espinosa N. **2018**. A review of the Cenozoic fossil record of the genus *Zamia* L. (Zamiaceae, Cycadales) with recognition of a new species from the late Eocene of Panama – evolution and biogeographic inferences. *Bulletin of Geosciences* **93**: 185–204.

Erdei B, Manchester SR. **2015**. *Ctenis clarnoensis* sp n., an unusual cycadalean foliage from the Eocene Clarno formation, Oregon. *International Journal of Plant Sciences* **176**: 31–43.

Erdei B, Manchester SR, Kvaček Z. **2012**. *Dioonopsis* Horiuchi et Kimura Leaves from the Eocene of Western North America: A Cycad Shared with the Paleogene of Japan. *International Journal of Plant Sciences* **173**: 81–95.

Feng Z, Lv Y, Guo Y, Wei H-B, Kerp H. **2017**. Leaf anatomy of a late Palaeozoic cycad. *Biology Letters* **13**: 20170456.

Florin R. **1933**. *Studien über die Cycadales des Mesozoikums, nebst Erörterungen über die Spaltöffnungsapparate der bennettitales*. Stockholm: Almqvist & Wiksells boktryckeri.

Harris TM. **1932**. *The Fossil Flora of Scoresby Sound, East Greenland: Part 2: Description of seed plants incertae sedis together with a discussion of certain Cycadophyta*. Copenhagen.

Harris TM. **1964**. *The Yorkshire Jurassic Flora. II. Caytoniales, Cycadales & Pteridosperms*. London: British Museum (Natural History).

Hill R. **1980**. Three new Eocene cycads from eastern Australia. *Australian Journal of Botany* **28**: 105.

Hill RS, Pole MS. **1994**. Two new species of *Pterostoma* R . S . Hill from Cenozoic sediments in Australasia. *Review of Palaeobotany and Palynology* **80**: 123–130.

Horiuchi J, Kimura T. **1987**. *Dioonopsis nipponica* gen. et. sp. nov., a new cycad from the palaeogene of Japan. *Review of Palaeobotany and Palynology* **51**: 213–225.

Krassilov VA. **1978**. Late Cretaceous gymnosperms from Sakhalin and the terminal Cretaceous event. *Palaeontology* **21**: 893–905.

Kvaček Z. **2002**. A new tertiary *Ceratozamia* (Zamiaceae, Cycadopsida) from the european Oligocene. *Flora* **197**: 303–316.

Kvaček J. **2008**. New Cycad Foliage of *Pseudoctenis babinensis* From the Bohemian Cenomanian. *Acta Musei Nationalis Pragae, Series B, Historia Naturalis* **64**: 125–131.

Kvaček Z. **2014**. New fossil records of *Ceratozamia* (Zamiaceae, Cycadales) from the European Oligocene and lower Miocene. *Acta Palaeobotanica* **54**: 231–247.

Kvaček Z, Manchester S. **1999**. *Eostangeria* Barthel (extinct cycadales) from the Paleogene of western North America and Europe. *International Journal of Plant Sciences* **160**: 621–629.

Passalia MG. **2013**. On the presence of the cycad *Pseudoctenis dentata* Archangelsky an d Baldoni in the Punta del Barco Formation (late Aptian), Santa Cruz Province, Argentina. *Ameghiniana* **50**: 257–264.

Pole M, Douglas B. **1999**. Plant Macrofossils of the Upper Cretaceous Kaitangata Coalfield, New Zealand. *Australian Journal of Botany* **12**: 331–364.

Pott C, Kerp JHF, Krings M. **2007**. *Pseudoctenis cornelii* nov. spec.(cycadalean foliage) from the Carnian (Upper Triassic) of Lunz, Lower Austria. *Annalen des Naturhistorischen Museums Wien* **109 A**: 1–17.

Su K, Quan C, Liu Y-S (Christopher). **2014**. *Cycas fushunensis* sp. nov. (Cycadaceae) from the Eocene of northeast China. *Review of Palaeobotany and Palynology* **204**: 43–49.

Uzunova K, Palamarev E, Kvaček Z. **2001**. *Eostangeria ruzinciniana* (Zamiaceae) from the Middle Miocene of Bulgaria and its relationship to similar taxa of fossil *Eostangeria*, and extant *Chigua* and *Stangeria* (Cycadales). *Acta Palaeobotanica* **41**: 177–193.

Van Konijnenburg-Van Cittert JHA, Van Der Burgh J. **1989**. The flora from the Kimmeridgian (upper jurassic) of Culgower, Sutherland, Scotland. *Review of Palaeobotany and Palynology* **61**: 1–51.

Watson J, Cusack HA. **2005**. Cycadales of the English Wealden. *Palaeontographical Society Monographs* **622**: 1–189.
